# Supplementary material for: Feasibility of a new electronic patient-reported outcome (ePRO) system for an advanced therapy clinical trial in immune-mediated inflammatory disease (PROmics): protocol for a qualitative feasibility study
Source: BMJ Open. 2022 Sep 6;12(9):e063199. doi: 10.1136/bmjopen-2022-063199 (PMC9453996; doi:10.1136/bmjopen-2022-063199)
Supplement: Supplementary data [file bmjopen-2022-063199supp001.pdf]

SUPPLEMENTAL FILE 1 – INTERVIEW TOPIC GUIDE

PATIENTS

1. Restate the purpose of the interview: *Discuss PROmics system*
2. Select the interview structure based on patient's ACCEPT, WITHDRAW, DECLINE status: If ACCEPT, ask ACCEPT questions only. If WITHDRAW, ask ACCEPT and WITHDRAW questions. If DECLINE, ask DECLINE questions only.
3. Any questions or clarification before beginning? Feel free to take a break at any point during the interview.
4. Materials: paper questionnaires, app with PROmics software for reference

ACCEPT

1. Why did you choose to use the PROmics software?
- Was there anything that made you question if you should use it? (FURTHER PROBES: Burden, confidence with technology)
- Were you given enough information?
2. Did you find the initial training with the PROmics software helpful?
- Was there anything particularly good or bad about the training?
- How could it be improved?
3. How did you find using the PROmics software?
- Did you feel like you had enough support?
- How could the support for patients using the PROmics software be improved?
4. How did use of the PROmics software fit in with your other trial-related activities? (FURTHER PROBE: Burden)
- Did it make you feel more or less engaged with the trial overall? How?
- Did it make you feel like you were getting better or worse care during the trial? How?
5. How did you find the PROmics software to use?
- What did you like? Why?
- What didn't you like? Why?
- How could it be improved?
- How does it compare to paper questionnaires? (SHOW: Paper questionnaires)
6. Would you recommend for others to use the PROmics software if they participating in a trial? Why?

WITHDRAW

1. Why did you decide to stop using the PROmics software?
- What could have been done to help you continue to use the software?

DECLINE

1. Why did you choose not to use the PROmics software?

- Was there anything that made you question if you should use it? (FURTHER PROBES: Burden, confidence with technology)
- Were you given enough information?

2. How does the PROmics software compare to paper questionnaires? (SHOW: app)

- Do you think you would use the PROmics software if you were in a trial again?

RESEARCH TEAM

1. Restate the purpose of the interview: *Discuss PROmics system*
2. Select the interview structure based on participant's experience: Site Research Nurse/ other. If OTHER, ask OTHER questions only. If SITE RESEARCH NURSE, ask SITE RESEARCH NURSE and OTHER questions.
3. Any questions or clarification before beginning? Feel free to take a break at any point during the interview.
4. Materials: paper questionnaires, demo iPad with PROmics software for reference

SITE RESEARCH NURSE

1. Did you find the initial training with the PROmics software helpful?

- Was there anything particularly good or bad about the training?
- How could it be improved?
- Were you given enough information?

2. How did you find using the PROmics software?

- Did you feel like you had enough support?
- How could the support for staff using the PROmics software be improved?

3. How did you find the PROmics software to use?

- What did you like? Why?
- What didn't you like? Why?
- How could it be improved?
- How does it compare to paper questionnaires? (SHOW: Paper questionnaires)

4. How did using the PROmics software impact on your care of patients?

- Was it better/worse?
- How could this be improved?

5. Did you feel equipped to train patients to use the PROmics software?

- How could this be improved?

6. Did you feel equipped to support patients to use the PROmics software during the trial?

- How could this be improved?

OTHER

1. How did use of the PROmics software fit in with your other trial-related activities? (FURTHER PROBES: Burden, workload)

- Was there anything that made you question if it should be used in the trial? (FURTHER PROBES: Burden, confidence with technology)
- Would you recommend that the PROmics software is used in future trials?
