# Supplementary material for: Feasibility of a new electronic patient-reported outcome (ePRO) system for an advanced therapy clinical trial in immune-mediated inflammatory disease (PROmics): protocol for a qualitative feasibility study
Source: BMJ Open. 2022 Sep 6;12(9):e063199. doi: 10.1136/bmjopen-2022-063199 (PMC9453996; doi:10.1136/bmjopen-2022-063199)
Supplement: Author's manuscript [file bmjopen-2022-063199.draft_revisions.pdf]

BMJ Open is committed to open peer review. As part of this commitment we make the peer review history of every article we publish publicly available.

When an article is published we post the peer reviewers' comments and the authors' responses online. We also post the versions of the paper that were used during peer review. These are the versions that the peer review comments apply to.

The versions of the paper that follow are the versions that were submitted during the peer review process. They are not the versions of record or the final published versions. They should not be cited or distributed as the published version of this manuscript.

BMJ Open is an open access journal and the full, final, typeset and author-corrected version of record of the manuscript is available on our site with no access controls, subscription charges or pay-per-view fees (http://bmjopen.bmj.com).

If you have any questions on BMJ Open's open peer review process please email info.bmjopen@bmj.com

BMJ Open

Protocol for a qualitative feasibility study of a new electronic patient-reported outcome (ePRO) system for an advanced therapy clinical trial in immune-mediated inflammatory disease: PROmics®

Journal:	*BMJ Open*
Manuscript ID	bmjopen-2022-063199
Article Type:	Protocol
Date Submitted by the Author:	27-Mar-2022

Complete List of Authors:	Hughes, Sarah; University of Birmingham, Centre for Patient Reported Outcome Research, Institute of Applied Health Research<br>McMullan, Christel; University of Birmingham, Centre for Patient Reported Outcome Research (CPROR), Institute of Applied Health Research<br>Rowe, Anna; University of Birmingham, National Institute for Health Research (NIHR) Biomedical Research Centre; University of Birmingham, Cancer Research UL Clinical Trials Unit<br>Retzer, Ameeta; University of Birmingham, Centre for Patient Reported Outcome Research<br>Malpass, Rebecca; University of Birmingham, Biomedical Research Centre; University of Birmingham, Cancer Research UK Clinical Trials Unit<br>Bathurst, Camilla; University of Birmingham, National Institute for Health Research (NIHR) Biomedical Research Centre; University of Birmingham, Cancer Research UK Clinical Trials Unit<br>Davies, Elin Haf; Aparito<br>Frost, Chris; Aparito<br>McNamara, Gary; Aparito<br>Harding, Rosie; University of Birmingham, Birmingham Law School<br>Price, Gary; University of Birmingham, Patient Partner, Centre for Patient Reported Outcome Research (CPROR)<br>Wilson, Roger; NCRI Consumer Forum, Sarcoma Patients Euronet<br>Walker, Anita; University of Birmingham, Centre for Patient Reported Outcome Research, Institute of Applied Health Research<br>Newsome, Philip; University of Birmingham, Institute of Immunology and Immunotherapy<br>Calvert , Melanie ; University of Birmingham, Centre for Patient Reported Outcomes Research and Institute of Applied Health Research

Keywords:	IMMUNOLOGY, Information technology < BIOTECHNOLOGY & BIOINFORMATICS, RHEUMATOLOGY, Hepatology < INTERNAL MEDICINE, Inflammatory bowel disease < GASTROENTEROLOGY

Protocol for a qualitative feasibility study of a new electronic patient-reported outcome (ePRO) system for an advanced therapy clinical trial in immune-mediated inflammatory disease: PROmics®

Sarah E Hughes,[1-3] Christel McMullan,[1,4,5] Anna Rowe,[6,7] Ameeta Retzer,[1,3,4] Rebecca Malpass,[6,7] Camilla Bathurst,[6,7] Elin Haf Davies,[8] Chris Frost,[8] Gary McNamara,[9] Rosie Harding,[10] Gary Price,[1] Roger Wilson,[11] Anita Walker,[1] Philip Newsome,[1,6,12,13] Melanie Calvert[1-4,6,13,14]

[1]Centre for Patient Reported Outcome Research (CPROR), Institute of Applied Health Research, University of Birmingham, Birmingham, UK
[2]National Institute for Health Research (NIHR) Applied Research Centre (ARC) West Midlands, Birmingham, UK
[3]Birmingham Health Partners Centre for Regulatory Science and Innovation, University of Birmingham, Birmingham, UK
[4]National Institute for Health Research (NIHR) Surgical Reconstruction and Microbiology Research Centre, University of Birmingham, Birmingham, UK
[5]Centre for Trauma Science Research, University of Birmingham, Birmingham, UK
[6]National Institute for Health Research (NIHR) Birmingham Biomedical Research Centre
[7]Cancer Research UK Clinical Trials Unit, University of Birmingham, Birmingham, UK
[8]Aparito Limited, Wrexham, UK
[9]Cognitant, UK
[10]Birmingham Law School, University of Birmingham, UK
[11]Patient Partner, Member of the National Cancer Research Institute (NCRI) Consumer Form, UK
[12]Institute of Immunology and Immunotherapy, University of Birmingham, Birmingham, UK
[13]Midlands Health Data Research UK, Birmingham, UK
[14]DEMAND Hub, University of Birmingham, Birmingham, UK

Address for correspondence:
Sarah Hughes
Centre for Patient Reported Outcome Research
Institute for Applied Health Research
University of Birmingham
Edgbaston
B15 2TT
s.e.hughes@bham.ac.uk
ORCID: 0000-0001-5656-1198

Word count: 3845 (excluding abstract, strengths and limitations, references, acknowledgments section)

For peer review only

For peer review only - http://bmjopen.bmj.com/site/about/guidelines.xhtml

ABSTRACT

Introduction The use of electronic patient-report outcome (ePRO) systems to capture PRO data in clinical trials is increasing; however, their feasibility, acceptability, and utility in clinical trials of advanced therapy medicinal products (ATMPs) are not yet well understood. This protocol describes a qualitative study to evaluate the feasibility and acceptability of ePRO data capture using an ePRO system within an advanced therapy trial involving patients with immune-mediated inflammatory disease.

Methods and analysis Semi-structured interviews will be conducted with 10-15 patients in enrolled in a single arm, phase II ATMP basket trial recruiting individuals with Rheumatoid Arthritis, Lupus, Primary Sclerosing Cholangitis and Crohn's Disease. Ten to fifteen research team members at the ATMP trial sites who are using the bespoke, trial-specific ePRO system (the PROmics® system) will also be interviewed. Interviews will be transcribed verbatim and analysed according to principles of thematic analysis. Data collection and analysis will occur concurrently and iteratively. Researcher triangulation will be used to achieve a consensus-based analysis, enhancing rigor and trustworthiness.

Ethics and dissemination This study was approved by the London - West London & GTAC Research Ethics Committee (Ref: 21/LO/0475). The study findings will be published in peer-review journals and disseminated via conference presentations and other media. Our patient and public involvement and engagement (PPIE) group and ATMP stakeholder networks will be consulted to maximise dissemination and impact.

For peer review only

For peer review only - http://bmjopen.bmj.com/site/about/guidelines.xhtml

STRENGTHS AND LIMITATIONS

- The use of an ePRO system to collect patient-reported outcome (PRO) data as part of an early-phase ATMP (advanced therapy medicinal product) clinical trial with patients with immune-mediated inflammatory disease could help to identify symptom burden and adverse side effects in research participants earlier, enhancing patient safety.
- This study will provide data on the feasibility and acceptability of the PROmics® approach to enhance future PRO strategy in later phases, promoting efficient use of measures; data completeness; acceptability to trial participants; and inform future analyses and sample size estimation.
- The study sample will include research participants from National Health Service (NHS) hospitals from different regions of the United Kingdom (UK) which may enhance the transferability and generalisability of the study findings.
- The study sample will include only those trial participants who consent to take part in this electronic PRO (ePRO) feasibility sub-study and therefore recruitment may be subject to selection bias.

For peer review only

For peer review only - http://bmjopen.bmj.com/site/about/guidelines.xhtml

INTRODUCTION

The use of stem cells, gene therapy and tissue engineering in the treatment of disease or injury represents an emerging and rapidly developing approach to medicine.[1 2] These advanced therapy medicinal products (ATMPs) have the potential to transform the treatment of some diseases (e.g., sickle cell disease, cancer, haemophilia), removing the need for long-term medical care. In the United Kingdom (UK) there has been steady year-on-year growth in the number of clinical trials of ATMPs.[3]

Endpoints in clinical trials are designed to assess the effects of an intervention, including the efficacy, safety and tolerability of the medicinal product. As an adjunct to clinical endpoint data, patient-reported outcome (PRO) data, when captured in a rigorous and scientific manner, can provide further, valuable evidence of treatment benefit or risk.[4 5] A PRO is "any report of the status of a patient's health condition that comes directly from the patient, without interpretation of the patient's response by a clinician or anyone else".[5] Such data include patient perspectives of symptom burden, functioning, and their health-related quality of life. In addition to their use as trial endpoints, PRO data may be used to support pharmaceutical labelling claims, clinical decision-making, clinical guideline development, and inform health policy.[6]

Historically, PROs have taken the form of self-completed, paper-based questionnaires that have been assessed psychometrically to establish their measurement properties. However, increasingly PRO instruments are being administered digitally as electronic Patient-Reported Outcomes (ePROs). ePRO systems typically use a digital device such as a computer, tablet or smartphone to deliver the PRO instrument(s) to the patient.[7] Leveraging remote connections and notification capability, ePROs can be programmed to trigger alerts to the clinical/trials staff should a participant report the presence of one or more symptoms of clinical concern.[8 9] In addition, ePROs offer the advantage of reducing data entry errors and respondent and administrative burden while facilitating data collection at specified time points and providing a record of the date and time entries are made.[7] Remote monitoring functionality enables trial participants to remain in their own homes at the same time as facilitating the surveillance of symptoms and disease status by the trial's clinical team.[8 10]

PROs in trials of ATMPs

Due to their novelty, complexity and technical specificity, ATMPs bring 'new, unexplored risks to patients', including risks such as unwanted immunogenicity and severe toxicity.[11] PROs may be used to directly capture symptomatic adverse events (AEs) and the overall treatment burden from the patient perspective and, therefore, have the potential to provide evidence on the safety and efficacy of an ATMP under investigation. In this way, PRO data can support post-treatment follow-up and risk management (including monitoring of the longer term effects on patients), provide essential data to inform real-world use, facilitate cost-benefit profiling, as well as support marketing authorisation.[2 12] In addition, PROs have potential to support a fuller understanding of tolerability, the ability or desire of the patient to adhere to a specific dose or intensity of therapy. Therefore, it is recommended that assessments of tolerability include direct measurements from the patient on how they are feeling and functioning while on treatment.[13 14]

The PROmics® ePRO system

The PROmics® system is an electronic data capture system designed to collect and assess PRO data when patients receive an advanced cell therapy. PROmics® captures PRO data relating to patient-reported adverse events and side effects, global tolerability of the medicinal product, and quality of life and daily functioning, tailored for each disease cohort. The PRO instruments selected for PROmics® were identified in collaboration with patient partners and other stakeholders in a workshop held in 2018. In addition to patient preference, selection was based upon regulatory recommendations, the psychometric properties of the PRO instruments, use in clinical practice and trials, and a need to ensure consistency in PRO reporting across disease cohorts (Table 1).

For peer review only - http://bmjopen.bmj.com/site/about/guidelines.xhtml

The following PRO instruments were selected for inclusion:

- The National Cancer Institute Patient-reported outcomes Version of the Common Terminology Criteria for Adverse Events (PRO-CTCAE®) is a 78-item questionnaire that has been developed to characterise the frequency, severity and interference of symptomatic treatment toxicities from the patient perspective. The PRO-CTCAE® was designed to be used as a companion to the CTCAE, the standard lexicon for AE reporting used in trials.[15-17]
- The FACT-G GP5 is a single item measuring patient-reported global treatment tolerability.[18]
- The EQ-5D-5L is a generic measure of self-rated health status. It consists of five items, each assessing a different health domain (mobility, self-care, usual activities, pain/discomfort, anxiety/depression), and a visual analogue scale (VAS) measuring global health status.[19]
- The FACIT-Fatigue scale is a 13-item generic PRO instrument measuring quality of life concerns relating to fatigue in chronic illness.[20 21]
- The Fatigue Severity Scale (FSS) is a nine-item generic instrument that assesses the severity of fatigue and its impact on daily living.[22]
- The Primary Sclerosing Cholangitis Patient Reported Outcomes (PSC-PRO) instrument is a 42-item questionnaire measuring PSC symptoms and their impact.[23]

Table 1: PRO instruments included in the PROmics® system for the POLARISE trial

PRO Instrument	Concept of interest	Disease cohort				Rationale for selection
		PSC	RA	LN	CD	
PRO-CTCAE	Burden (frequency, severity, interference) of symptomatic toxicities	X	X	X	X	Side effect and adverse event monitoring
FACT-G GP5	Global tolerability of treatment	X	X	X	X	Monitoring of treatment tolerability from the patient perspective
EuroQol 5D-5L (EQ5D-5L)	Health status	X	X	X	X	Local health reimbursement requirement
Fatigue Severity Scale (FSS)	Fatigue severity and impact on daily living	X	X	X	X	Preferred by PSC patients, use in previous trials, methodological consistency and to maximise comparability between trial arms
FACIT-Fatigue	Quality of life rating to fatigue	X	X	X	X	Recommended for use in RA patients by the European Medicines Association (EMA) and the International Consortium for Health Outcomes Measurement (ICHOM), methodological consistency, maximise comparability between trial arms
PSC-PRO	PSC symptom severity and impact	X				PSC-specific PRO instrument, patient preference, instrument developed with extensive patient input

CD = Crohn's Disease; LN = Lupus Nephritis; RA = Rheumatoid Arthritis; PSC = Primary Sclerosing Cholangitis

To complete the PRO instruments, patients access the PROmics® system via an app on their personal tablet or smartphone, a data collection system known as "Bring Your Own Device (BYOD)".[7] The PROs

For peer review only - http://bmjopen.bmj.com/site/about/guidelines.xhtml

are delivered in a standardised ordered for self-completion by the trial participant. Pre-programmed notifications will remind participants to submit their completed questionnaires, if they have not done so within a specified time-period. Clinical alerts, in the form of email notifications to the research site team, are triggered if a participant reports a high symptom burden. The alert, in the form of an email message to named trial staff at the relevant research site, prompts staff to login to the PROmics® clinical dashboard, review the PRO scores for the corresponding trial ID number, and, if required, complete an adverse event (AE) report using the Common Terminology Criteria for Adverse Events (CTCAE).[24]

POLARISE Trial

The PROmics feasibility study is set in the context of the POLARISE Trial (ISRCTN80103507), a single arm, multi-centre, phase II basket trial investigating the safety and activity of ORBCEL-C® in the treatment of patients with immune-mediated inflammatory disease – specifically, Primary Sclerosing Cholangitis (PSC), Rheumatoid Arthritis (RA), Lupus Nephritis (LN) and Crohn's Disease (CD). These disease groups represent a highly heterogeneous sample in terms of age, physical impairment, and other disease-related characteristics. However, all are associated with high symptom burden and reduced quality of life (QoL), necessitating the collection of PROs at scheduled time-points as well as notifications of patient-reported adverse events (AEs) when symptoms of clinical concern are reported by participants. Remote collection of PRO data is possible through use of an ePRO system, with its specific mode of delivery and enhanced functionality, enabling these trial specific requirements to be met.

AIMS AND OBJECTIVES

To qualitatively assess the feasibility and acceptability of the PROmics® system, an ePRO platform for use in trials of ATMPs. The planned study will evaluate the use of PROmics® when deployed in a trial environment generally and, more specifically, in the POLARISE study, a phase II multi-disease ATMP trial in patients with immune-mediated inflammatory disease.

The specific study objectives are:

1. To explore with PROmics® users, their' experiences of being trained with and using the PROmics® ePRO system;
2. To explore decision-making with POLARISE trial participants who did not consent to report their PROs via PROmics® or withdrew from using the PROmics®;
3. To explore the benefits and shortcomings of PROmics® and how these may be developed further;
4. To explore how the use of PROmics® was managed by research staff and research participants in the context of the POLARISE trial.

METHODS AND ANALYSIS

This study will be reported in accordance with the Consolidated Criteria for Reporting Qualitative Research (COREQ).[25]

Design

This feasibility assessment, embedded in the POLARISE trial, will use qualitative methods to elicit the experiences and perspectives of participants regarding user experience, product perception, compliance, acceptability, and feasibility of use.

Participant selection

In total, it is estimated that 20 to 30 participants will be recruited to this feasibility study. Recruitment will proceed until data saturation, defined as the degree to which new data express what was expressed

For peer review only - http://bmjopen.bmj.com/site/about/guidelines.xhtml

in previous data, is achieved.[26] Two participant groups, POLARISE trial participants (n = 10 – 15 participants) and members of the POLARISE research team (n = 10-15 participants) will be enrolled.

Sampling will be purposive for maximum variation for both POLARISE participants and research staff. Maximum variation where possible will be sought to ensure broad representation of disease groups, age, sex, ethnicity, research site/geographic location (POLARISE participants) and age, sex, clinical/research role, research site/geographic location (research staff).

To take part in the PROmics® feasibility study, patients must be enrolled in the POLARISE trial (therefore meeting the trial eligibility criteria) and have: 1) consented to report their PRO data (symptoms, tolerability, quality of life) using the PROmics® ePRO system; 2) consented initially to report their PROs using the PROmics® and subsequently withdrew from using the PROmics® system to report their PRO data, reverting to paper questionnaires; or 3) declined to report their PROs using the PROmics® software . Sampling will aim to recruit between three and five participants from each disease group and any POLARISE participants who decline to use or subsequently withdraw from using the PROmics® system.

Participants who are research staff will be members of the research team who have been involved in the planning, delivery, administration and use of the PROmics® ePRO system including: 1) research nurses working on the POLARISE trial; 2) POLARISE trial staff; and 3) staff involved in the management of POLARISE trial data.

All PROmics® participants must be able to converse in everyday English and be 18 years old or older. Exclusion criteria include being unable to read, understand and complete questionnaires presented in English, and declining the optional consent for the use of the PROmics system.

Recruitment and consent

All POLARISE patient participants will be eligible to take part in this feasibility study and will be provided with information packs relating to the feasibility study upon providing their consent to take part in the POLARISE trial. The information packs will be tailored depending on whether a participant has: 1) consented to report their PRO data via the PROmics® system (accept); 2) consented to provide PRO data via PROmics® and changed to paper-based PROs (withdraw); or 3) declined to report their PRO data via the PROmics® software (decline). All versions (accept/withdraw/decline) of the information pack will include a cover letter, participant information sheet, and an expression of interest form (+ reply paid envelope) to be returned by the participant to the PROmics® research team should they wish to take part in the feasibility study.

Research team participants will be approached and provided with recruitment packs via liaison with the POLARISE trial sites. The recruitment packs will include a cover letter, participant information sheet, and expression of interest form.

A semi-structured interview conducted via telephone or videoconference (e.g., Zoom) will be arranged with all those participants (patients and members of the POLARISE research team) who expressed an interest in participating in the feasibility study. Verbal consent will be sought at the time of the telephone interview. A pre-specified consent script will enable consent to be taken via the telephone. Participants will be reminded of the aim of the study and that they are able to withdraw from the study at any time. The consenting process will be completed prior to commencing the interview and audio recorded.

Data collection

Once participants have been enrolled into the POLARISE trial, the telephone interviews will be scheduled. Interviews will be arranged at a time point in the trial to allow participants sufficient opportunity to use the PROmics® system and to ensure accurate recall of their experiences whilst

For peer review only - http://bmjopen.bmj.com/site/about/guidelines.xhtml

enrolled in the POLARISE trial. As the POLARISE trial is a national trial with multiple sites, this method of data collection was selected to enable: 1) ease of communication between participants and the PROmics® research team and 2) ensure safe social distancing for COVID-19.

Semi-structured topic guides will support patient and research staff interviews and will include set/specific questions for participants regarding the selection, perception, experiences, feasibility and acceptability of the PROmics® system and will ensure that key topics are consistently covered. The semi-structured nature of the planned interviews will enable the researcher conducting the interviews to explore topics in depth and accommodate emerging themes through prompts and questions that are informed by the participants' responses. All interviews will be audio-recorded using a digitally encrypted recorder, transcribed verbatim by a qualified, professional transcription service working within a confidentiality agreement. To confirm the accuracy of the interview transcripts, the transcripts will be reviewed against the audio-recording by the PROmics® research team. All transcripts will be de-identified and a unique identifier will be assigned to each participant prior to undertaking data analyses. Data collection and analysis will take place concurrently and will continue until the research team agrees data saturation has been reached (per participant group and overall).

Data analysis

The de-identified interview transcripts will be entered into NVivo (Version 20) qualitative data analysis software and analysed thematically to identify, describe and report patterns in the data.[27 28] Each participant group (i.e., patients and research staff) will be analysed separately and then compared later in the analysis to produce a rich, nuanced description of the full data set. Initially, transcripts will be coded inductively line-by-line to develop a set of initial codes. Initial codes will be examined and grouped into higher-level categories (themes) which, in turn, will be refined to ensure they encompass the full data set and present a clear, coherent and detailed interpretation of the data. Data will be analysed by two researchers (CM and SEH) who will compare findings to produce a final consensus-based analysis. The final analysis will be discussed with the POLARISE trial Patient and Public Involvement and Engagement (PPIE) group to establish the trustworthiness and validity of the findings.

Reflexivity

Reflexivity may be defined as researchers' acknowledgment of their own beliefs, judgements and practices and the role these play during the research process.[29] It is central to conducting a thematic analysis as the researcher plays an active role in the identification patterns within the data.[27 28] The PROmics® research team comprises experts in PROs (MC, SEH, CM), qualitative research (CM, SEH), clinical trials (AR, RM), ePROs (CF, EHD), and a consultant hepatologist (PN). It is nested within the larger POLARISE trial research team. Two experienced qualitative researchers (CM, SEH) will conduct the interviews and undertake data analysis. None of the research team have any relationship with patient participants; however, it is possible that participants who are research staff at POLARISE trial sites may be known contacts to some members of the PROmics® team.

PATIENT AND PUBLIC INVOLVEMENT AND ENGAGEMENT

PPIE in the PROmics® study and wider POLARISE trial was informed by the National Institute for Health Research (NIHR) guidance on the involvement of patient public contributors to research.[30] Patient partners were involved from the outset of the project. To date, PPIE members have informed the design and content of the PROmics® ePRO system. A series of stakeholder meetings were convened to establish patient preferences for the format and layout of the PROmics® system and to inform PRO selection. Prior to deployment in the POLARISE trial, usability testing of the PROmics® system was completed with PPIE members. The outcomes of the stakeholder engagement events, and usability testing are reported elsewhere. This protocol was reviewed by patient partner (RW) and the Liver and Gastro-Intestinal PPI Reference Group at the University of Birmingham and the Birmingham

For peer review only - http://bmjopen.bmj.com/site/about/guidelines.xhtml

Rheumatology Research Patient Partnership (R2P2) who will also support the dissemination of information about the study. Patient and public involvement will be evaluated at the end of the project using the GRIPP-2 checklist.[31 32]

ETHICS AND DISSEMINATION

Ethical approval for the PROmics® feasibility study was granted as part of the POLARISE trial by London - West London & GTAC Research Ethics Committee (21/LO/0475). Local R&D approvals will be obtained prior to recruitment at sites. The results of this study will be disseminated via peer-reviewed journals, conference presentations and shared via social media. The PPIE groups and ATMP stakeholder networks will be consulted to maximise dissemination and impact.

DISCUSSION

Clinical trialists, regulators and policy-makers recognise increasingly the importance of the patient experience in the evaluation of the effectiveness, safety, and tolerability of medicinal products, including in trials of advanced cellular and gene therapies.[2 33] Accordingly, PRO assessments are included in clinical trials to measure constructs such as symptom severity and functioning and health-related quality of life and, more and more, these data are collected using ePROs. However, the impact on patients and the feasibility and acceptability of ePRO data capture has yet to be established fully in ATMP trials. This qualitative study aims to provide a deeper understanding into the use ePROs in clinical trials of advanced therapies. In particular, barriers and enablers to the use of PROmics®, a new ePRO data capture system, will be explored within an ATMP trial for patients with immune-mediated inflammatory disease. These qualitative data will be supplemented with further findings at the end of the POLARISE trial including compliance and rates of missing data. The findings will inform recommendations to improve the PROmics® system, to enhance future PRO strategy in future studies, promote efficient use of measures; data completeness; acceptability to trial participants; and inform future analyses and sample size estimation, and to support future implementation of ePROs in ATMP trials and routine care.

The study will incorporate the views of a range of stakeholders. The involvement of trial participants from different disease cohorts and from different geographical locations in the UK will enhance variation in the study sample. These patient participants will provide insights into user confidence with digital technology and the completion of PROs electronically, time points of administration, and the overall user experience. Inclusion of trial participants who declined or withdrew from using the PROmics® will allow us to explore potential barriers and solutions to participation including inclusion of underserved populations. The views of trial staff and site research nurses will help understand PROmics® integration with local IT services and the administrative burden on staff as well as barriers to and enablers of the use of PROmics® for data collection, trial participant surveillance, and AE reporting,

The use of qualitative methods is a strength of this study, allowing in-depth exploration of user experience. Nesting the feasibility study within the POLARISE trial will enable timely data collection thereby limiting recall bias. The study sample will include research participants from NHS hospitals from different regions of the UK which may enhance the transferability and generalisability of the study findings. However, as not all trial participants will take part in the feasibility study, sampling is potentially biased. Sampling bias will be addressed by recruiting purposively for maximum variation and through the inclusion of participants who declined to use the PROmics system or who reverted to the use of paper-based PROs at some point in the trial in the study sample. Questionnaires programmed onto the PROmics® system are currently in UK English, however, if the solution proves feasible, inclusion of translated, culturally validated measures will be undertaken in future work.

For peer review only - http://bmjopen.bmj.com/site/about/guidelines.xhtml

The number of ATMP trials continues to grow year-on-year; offering potentially powerful new treatments for patients with rare or life-threatening diseases.[34] As with all investigational medicinal products, demonstrating efficacy, safety and tolerability of new ATMPs is crucial. Electronic PROs have the potential to contribute important evidence of efficacy, safety and tolerability (both in the short and longer term) for these novel therapies and support patient monitoring and risk management. Data collected via the PROmics® system has the potential to provide new insights into the reporting of symptomatic AEs specific to the advanced therapies context whilst evaluation of the feasibility and acceptability of the PROmics® system will contribute new knowledge to support the development of ePRO reporting formats that meet the unique needs of advanced therapies, for the benefit of all who receive these novel treatments.

ACKNOWLEDGMENTS

The authors thank POLARISE Trial Steering Committee, POLARISE Scientific Advisory Group, PROmics Project Management Group, and Patient and Public Involvement (PPI) group members for their input and support of the project.

Contributors
The feasibility study concept and design were conceived by AmR, PN, MC, RW, RH, GP. CB, AR will support recruitment. EHD, CF will support POLARISE data collection using the PROmics® system. SEH and CM will consent participants and undertake the interviews and data analysis. The protocol was written by AmR, MC, AR, PN. SEH prepared the first draft of the manuscript. All authors (SEH, CM AR, AmR, RM, CB, EHD, CF, GM, RH, GP, RW, PN, MC) reviewed the manuscript, provided edits and approved the final version.

Funding
The author(s) disclosed receipt of the following financial support for the research, authorship, and/or publication of this article: This work was supported by the National Institute for Health Research (NIHR) Birmingham Biomedical Research Centre, Midlands-Wales Advanced Therapies Treatment Centre (MW-ATTC) programme grant from Innovate UK to a consortium of partners including Health Technology Wales, the Welsh Blood Service and the University of Birmingham (Grant number: IUK: 104232) and Innovate UK (part of UK Research and Innovation) grant Patient-reported outcomes assessment to support accelerated access to advanced cell and gene therapies: PROmics (Grant No: IUK: 104777). The funder was not involved in any aspect of the research work.

Competing Interests
SEH receives funding from the National Institute for Health Research (NIHR) Applied Research Centre (ARC), West Midlands at the University of Birmingham and UK Research and Innovation (UKRI). SEH declares personal fees from Cochlear Ltd and Aparito Ltd outside the submitted work. *MC* is a National Institute for Health Research (NIHR) Senior Investigator and receives funding from the National Institute for Health Research (NIHR) Birmingham Biomedical Research Centre, the NIHR Surgical Reconstruction and Microbiology Research Centre and NIHR ARC West Midlands at the University of Birmingham and University Hospitals Birmingham NHS Foundation Trust, Health Data Research UK, UKRI, Macmillan Cancer Support, UCB Pharma Gilead, Janssen and GSK. MC has received personal fees from Astellas, Aparito Ltd, CIS Oncology, Takeda, Merck, Daiichi Sankyo, Glaukos, GSK and the Patient-Centered Outcomes Research Institute (PCORI) outside the submitted work. *CM* receives funding from NIHR Surgical Reconstruction and Microbiology Research Centre (SRMRC), UKRI, and declares personal fees from Aparito Ltd outside the submitted work. All other authors have no interests to declare.

Disclaimer
The views expressed are those of the authors and not necessarily those of Innovate UK.

Patient consent for publication
Not required.

Provenance and peer review
Not commissioned, externally peer reviewed.

Open access
This is an open access article distributed in accordance with the Creative Commons Attribution 4.0 International license (CC BY 4.0), which permits others to distribute, remix, adapt, build upon this work, and license their derivative works on different terms, provided the original work is properly cited,

For peer review only - http://bmjopen.bmj.com/site/about/guidelines.xhtml

appropriate credit is given, and any changes made indicated. See: http:// https://creativecommons.org/licenses/by/4.0/.

REFERENCES

1. Elverum K, Whitman M. Delivering cellular and gene therapies to patients: solutions for realizing the potential I'of the next generation of medicine. *Gene Ther* 2020;27:537-44. doi: 10.1038/s41434-019-0074-7
2. Lasiter L, Campbell A, Basch E, et al. Use of Patient-Reported Outcomes to Understand & Measure the Patient Experience of Novel Cell and Gene Therapies. *Ther Innov Regul Sci* 2020;54:1566-75. doi: 10.1007/s43441-020-00184-6
3. Hollingsworth I. Increasing patient access to advanced therapies. *International Biopharmaceutical Industry* 2020; 3(1). https://www.theattcnetwork.co.uk/news/increasing-patient-access-to-advanced-therapies-the-uk-perspective (accessed 25 October 2021).
4. Kyte D, Ives J, Draper H, et al. Current practices in patient-reported outcome (PRO) data collection in clinical trials: a cross-sectional survey of UK trial staff and management. *BMJ Open* 2016;6:e012281. doi: 10.1136/bmjopen-2016-012281
5. US Food and Drug Administration. Guidance for industry: Patient-reported outcome measures: Use in medicinal product development to support labelling claims, 2009. https://www.fda.gov/regulatory-information/search-fda-guidance-documents/patient-reported-outcome-measures-use-medical-product-development-support-labeling-claims (accessed 25 October 2021)
6. Calvert M, Kyte D, Mercieca-Bebber R, et al. Guidelines for Inclusion of Patient-Reported Outcomes in Clinical Trial Protocols: The SPIRIT-PRO Extension. *JAMA* 2018;319:483-94. doi: 10.1001/jama.2017.21903
7. Coons SJ, Eremenco S, Lundy JJ, et al. Capturing Patient-Reported Outcome (PRO) Data Electronically: The Past, Present, and Promise of ePRO Measurement in Clinical Trials. *Patient*. 2015 Aug;8:301-9. doi: 10.1007/s40271-014-0090-z. Erratum in: *Patient*. 2015 Dec;8(6):571.
8. Holch P, Pini S, Henry AM, et al. eRAPID electronic patient self-Reporting of Adverse-events: Patient Information and aDvice: a pilot study protocol in pelvic radiotherapy. *Pilot Feasibility Stud* 2018;4:110. doi: 10.1186/s40814-018-0304-6
9. Kyte D, Ives J, Draper H, et al. Management of Patient-Reported Outcome (PRO) Alerts in Clinical Trials: A Cross Sectional Survey. *PloS One* 2016;11:e0144658-e58. doi: 10.1371/journal.pone.0144658
10. Basch E, Deal AM, Kris MG, et al. Symptom Monitoring With Patient-Reported Outcomes During Routine Cancer Treatment: A Randomized Controlled Trial. *J Clin Oncol* 2016;34:557-65. doi: 10.1200/JCO.2015.63.0830
11. European Medicines Agency. Guidline on Safety and Efficacy Follow-Up - Risk Management of Advanced Therapy Medicinal Products. 2008. https://www.ema.europa.eu/en/documents/scientific-guideline/guideline-safety-efficacy-follow-risk-management-advanced-therapy-medicinal-products_en.pdf (accessed 25 October 2021).
12. Calvert M, Kyte D, Price G, et al. Maximising the impact of patient reported outcome assessment for patients and society. *BMJ* 2019;364:k5267. doi: 10.1136/bmj.k5267
13. Kluetz PG, Kanapuru B, Lemery S, et al. Informing the Tolerability of Cancer Treatments Using Patient-Reported Outcome Measures: Summary of an FDA and Critical Path Institute Workshop. *Value Health* 2018;21:742-47. doi: https://doi.org/10.1016/j.jval.2017.09.009
14. European Medicines Agency. Appendix 2 to the guideline on the evaluation of anticancer medicinal products in man: The use of patient-reported outcome (PRO) measures in oncology studies. 2016. https://www.ema.europa.eu/en/documents/other/appendix-2-guideline-evaluation-anticancer-medicinal-products-man_en.pdf (accessed 25 October 2021).

For peer review only - http://bmjopen.bmj.com/site/about/guidelines.xhtml

15. Smith AW, Mitchell SA, K. De Aguiar C, et al. News from the NIH: Person-centered outcomes measurement: NIH-supported measurement systems to evaluate self-assessed health, functional performance, and symptomatic toxicity. *Transl Behav Med* 2016;6:470-74. doi: 10.1007/s13142-015-0345-9
16. Kluetz PG, Chingos DT, Basch EM, et al. Patient-Reported Outcomes in Cancer Clinical Trials: Measuring Symptomatic Adverse Events With the National Cancer Institute's Patient-Reported Outcomes Version of the Common Terminology Criteria for Adverse Events (PRO-CTCAE). *Am Soc Clin Oncol Educ Book* 2016;35:67-73. doi: 10.1200/EDBK_159514
17. Dueck AC, Mendoza TR, Mitchell SA, et al. Validity and Reliability of the US National Cancer Institute's Patient-Reported Outcomes Version of the Common Terminology Criteria for Adverse Events (PRO-CTCAE). *JAMA Oncol* 2015;1:1051-59. doi: 10.1001/jamaoncol.2015.2639
18. Pearman TP, Beaumont JL, Mroczek D, et al. Validity and usefulness of a single-item measure of patient-reported bother from side effects of cancer therapy. *Cancer* 2018;124:991-97. doi: https://doi.org/10.1002/cncr.31133
19. Herdman M, Gudex C, Lloyd A, et al. Development and preliminary testing of the new five-level version of EQ-5D (EQ-5D-5L). *Qual Life Res* 2011;20:1727-36. doi: 10.1007/s11136-011-9903-x
20. Cella DF, Tulsky DS, Gray G, et al. The Functional Assessment of Cancer Therapy scale: development and validation of the general measure. *J Clin Oncol* 1993;11:570-79. doi: 10.1200/jco.1993.11.3.570
21. Webster K, Cella D, Yost K. The Functional Assessment of Chronic Illness Therapy (FACIT) Measurement System: properties, applications, and interpretation. *Health Qual Life Outcomes* 2003;1:79. doi: 10.1186/1477-7525-1-79
22. Krupp LB, LaRocca NG, Muir-Nash J, et al. The Fatigue Severity Scale: Application to Patients With Multiple Sclerosis and Systemic Lupus Erythematosus. *Arch Neurol* 1989;46:1121-23. doi: 10.1001/archneur.1989.00520460115022
23. Younossi ZM, Afendy A, Stepanova M, et al. Development and validation of a primary sclerosing cholangitis–specific patient-reported outcomes instrument: The PSC PRO. *Hepatology* 2018;68:155-65. doi: https://doi.org/10.1002/hep.29664
24. National Cancer Institute. Common Terminology Criteria for Adverse Events (CTCAE) v4.0: NCI; [Available from: https://evs.nci.nih.gov/ftp1/CTCAE/CTCAE_4.03/CTCAE_4.03_2010-06-14_QuickReference_5x7.pdf accessed 26 October 2021).
25. Tong A, Sainsbury P, Craig J. Consolidated criteria for reporting qualitative research (COREQ): a 32-item checklist for interviews and focus groups. *Int J Qual Health Care* 2007;19:349-57. doi: 10.1093/intqhc/mzm042
26. Saunders B, Sim J, Kingstone T, et al. Saturation in qualitative research: exploring its conceptualization and operationalization. *Qual Quant* 2018;52:1893-907. doi: 10.1007/s11135-017-0574-8
27. Braun V, Clarke V. Using thematic analysis in psychology. *Qual Res Psychol* 2006;3:77-101. doi: 10.1191/1478088706qp063oa
28. Braun V, Clarke V. Reflecting on reflexive thematic analysis. *Qual Res Sport, Exerc Health* 2019;11:589-97. doi: 10.1080/2159676X.2019.1628806
29. Finlay L. Reflexivity: An Essential Component for All Research? *Br J Occup Ther* 1998;61:453-56. doi: 10.1177/030802269806101005
30. National Institute for Health Research. Centre for Dissemination and Engagement recognition payments for public contributors 2020 [9 January 2021]. Available from: https://www.nihr.ac.uk/documents/centre-for-engagement-and-dissemination-recognition-payments-for-public-contributors/24979 (accessed 25 October 2021).
31. Staniszewska S, Brett J, Simera I, et al. GRIPP2 reporting checklists: tools to improve reporting of patient and public involvement in research. *Res Involv Engagem* 2017;3:13. doi: 10.1186/s40900-017-0062-2

For peer review only - http://bmjopen.bmj.com/site/about/guidelines.xhtml

32. Kok M, Gibson A, Evans D, et al. Guidance document: Evaluating public involvement in research. 2018. http://www.phwe.org.uk/wp-content/uploads/Guidance-on-evaluating-Public-Involvement-in-research.pdf (accessed 26 October 2021).
33. Chakraborty R, Sidana S, Shah GL, et al. Patient-Reported Outcomes with Chimeric Antigen Receptor T Cell Therapy: Challenges and Opportunities. *Biol Blood Marrow Transplant* 2019;25:e155-e62. doi: https://doi.org/10.1016/j.bbmt.2018.11.025
34. Hanna E, Rémuzat C, Auquier P, et al. Advanced therapy medicinal products: current and future perspectives. *J Mark Access Health Policy* 2016;4:10.3402/jmahp.v4.31036. doi: 10.3402/jmahp.v4.31036

For peer review only

For peer review only - http://bmjopen.bmj.com/site/about/guidelines.xhtml

BMJ Open

Feasibility of a new electronic patient-reported outcome (ePRO) system for an advanced therapy clinical trial in immune-mediated inflammatory disease (PROmics): protocol for a qualitative feasibility study

Journal:	*BMJ Open*
Manuscript ID	bmjopen-2022-063199.R1
Article Type:	Protocol
Date Submitted by the Author:	18-Jul-2022

Complete List of Authors:	Hughes, Sarah; University of Birmingham, Centre for Patient Reported Outcome Research, Institute of Applied Health Research<br>McMullan, Christel; University of Birmingham, Centre for Patient Reported Outcome Research (CPROR), Institute of Applied Health Research<br>Rowe, Anna; University of Birmingham, National Institute for Health Research (NIHR) Biomedical Research Centre; University of Birmingham, Cancer Research UL Clinical Trials Unit<br>Retzer, Ameeta; University of Birmingham, Centre for Patient Reported Outcome Research<br>Malpass, Rebecca; University of Birmingham, Biomedical Research Centre; University of Birmingham, Cancer Research UK Clinical Trials Unit<br>Bathurst, Camilla; University of Birmingham, National Institute for Health Research (NIHR) Biomedical Research Centre; University of Birmingham, Cancer Research UK Clinical Trials Unit<br>Davies, Elin Haf; Aparito<br>Frost, Chris; Aparito<br>McNamara, Gary; Aparito<br>Harding, Rosie; University of Birmingham, Birmingham Law School<br>Price, Gary; University of Birmingham, Patient Partner, Centre for Patient Reported Outcome Research (CPROR)<br>Wilson, Roger; NCRI Consumer Forum, Sarcoma Patients Euronet<br>Walker, Anita; University of Birmingham, Centre for Patient Reported Outcome Research, Institute of Applied Health Research<br>Newsome, Philip; University of Birmingham, Institute of Immunology and Immunotherapy<br>Calvert , Melanie ; University of Birmingham, Centre for Patient Reported Outcomes Research and Institute of Applied Health Research

**Primary Subject Heading**:	Immunology (including allergy)
Secondary Subject Heading:	Health services research
Keywords:	IMMUNOLOGY, Information technology < BIOTECHNOLOGY & BIOINFORMATICS, RHEUMATOLOGY, Hepatology < INTERNAL MEDICINE, Inflammatory bowel disease < GASTROENTEROLOGY

SCHOLARONE™
Manuscripts

Feasibility of a new electronic patient-reported outcome (ePRO) system for an advanced therapy clinical trial in immune-mediated inflammatory disease (PROmics): protocol for a qualitative feasibility study

Sarah E Hughes,[1-3,15] Christel McMullan,[1,4,5,15] Anna Rowe,[6,7] Ameeta Retzer,[1,3,4] Rebecca Malpass,[6,7] Camilla Bathurst,[6,7] Elin Haf Davies,[8] Chris Frost,[8] Gary McNamara,[9] Rosie Harding,[10] Gary Price,[1] Roger Wilson,[11] Anita Walker,[1] Philip Newsome,[1,6,12,13] Melanie Calvert[1-4,6,13,14,15]

[1]Centre for Patient Reported Outcome Research (CPROR), Institute of Applied Health Research, University of Birmingham, Birmingham, UK
[2]National Institute for Health Research (NIHR) Applied Research Centre (ARC) West Midlands, Birmingham, UK
[3]Birmingham Health Partners Centre for Regulatory Science and Innovation, University of Birmingham, Birmingham, UK
[4]National Institute for Health Research (NIHR) Surgical Reconstruction and Microbiology Research Centre, University of Birmingham, Birmingham, UK
[5]Centre for Trauma Science Research, University of Birmingham, Birmingham, UK
[6]National Institute for Health Research (NIHR) Birmingham Biomedical Research Centre
[7]Cancer Research UK Clinical Trials Unit, University of Birmingham, Birmingham, UK
[8]Aparito Limited, Wrexham, UK
[9]Cognitant, UK
[10]Birmingham Law School, University of Birmingham, UK
[11]Patient Partner, Member of the National Cancer Research Institute (NCRI) Consumer Form, UK
[12]Institute of Immunology and Immunotherapy, University of Birmingham, Birmingham, UK
[13]Midlands Health Data Research UK, Birmingham, UK
[14]DEMAND Hub, University of Birmingham, Birmingham, UK
[15]NIHR Oxford-Birmingham Blood and Transplant Research Unit (BTRU) in Precision Therapeutics

Address for correspondence:
Sarah Hughes
Centre for Patient Reported Outcome Research
Institute for Applied Health Research
University of Birmingham
Edgbaston
B15 2TT
s.e.hughes@bham.ac.uk
ORCID: 0000-0001-5656-1198

Word count: 4082 words (Excluding tables, acknowledgements, and references)

For peer review only - http://bmjopen.bmj.com/site/about/guidelines.xhtml

ABSTRACT

Introduction The use of electronic patient-report outcome (ePRO) systems to capture PRO data in clinical trials is increasing; however, their feasibility, acceptability, and utility in clinical trials of advanced therapy medicinal products (ATMPs) are not yet well understood. This protocol describes a qualitative study that aims to evaluate the feasibility and acceptability of ePRO data capture using a bespoke, trial-specific ePRO system (the PROmics system) within an advanced therapy trial involving patients with immune-mediated inflammatory disease (Rheumatoid Arthritis, Lupus, Primary Sclerosing Cholangitis and Crohn's Disease).

Methods and analysis 10-15 patients enrolled in the POLARISE trial, a single arm, phase II ATMP basket trial, and 10-15 research team members at the ATMP trial sites will be recruited. Participants will take part in semi-structured interviews which will be transcribed verbatim and analysed thematically according to the framework method. Data collection and analysis will occur concurrently and iteratively. Researcher triangulation will be used to achieve a consensus-based analysis, enhancing rigor and trustworthiness.

Ethics and dissemination This study was approved by the London - West London & GTAC Research Ethics Committee (Ref: 21/LO/0475). The study findings will be published in peer-review journals and disseminated via conference presentations and other media. Our patient and public involvement and engagement (PPIE) group and ATMP stakeholder networks will be consulted to maximise dissemination and impact.

For peer review only

STRENGTHS AND LIMITATIONS

- The use of an ePRO system to collect patient-reported outcome (PRO) data as part of an early-phase ATMP (advanced therapy medicinal product) clinical trial with patients with immune-mediated inflammatory disease could help to identify symptom burden and adverse side effects in research participants earlier, enhancing patient safety.
- This study will provide data on the feasibility and acceptability of the PROmics approach to enhance future PRO strategy in later phases, promoting efficient use of measures; data completeness; acceptability to trial participants; and inform future analyses and sample size estimation.
- The study sample will include research participants from National Health Service (NHS) hospitals from different regions of the United Kingdom (UK) which may enhance the transferability and generalisability of the study findings.
- The study sample will include only those trial participants who consent to take part in this electronic PRO (ePRO) feasibility sub-study and therefore recruitment may be subject to selection bias.

For peer review only

For peer review only - http://bmjopen.bmj.com/site/about/guidelines.xhtml

INTRODUCTION

The use of stem cells, gene therapy and tissue engineering in the treatment of disease or injury represents an emerging and rapidly developing approach to medicine.[1, 2] These advanced therapy medicinal products (ATMPs) have the potential to transform the treatment of some diseases (e.g., sickle cell disease, cancer, haemophilia), removing the need for long-term medical care. In the United Kingdom (UK) there has been steady year-on-year growth in the number of clinical trials of ATMPs.[3]

Endpoints in clinical trials are designed to assess the effects of an intervention, including the efficacy, safety and tolerability of the medicinal product. As an adjunct to clinical endpoint data, patient-reported outcome (PRO) data, when captured in a rigorous and scientific manner, can provide further, valuable evidence of treatment benefit or risk.[4, 5] A PRO is "any report of the status of a patient's health condition that comes directly from the patient, without interpretation of the patient's response by a clinician or anyone else".[5] PRO data include patient perspectives of symptom burden, functioning, and their health-related quality of life and are typically captured using validated, self-report questionnaires. PROs may be used alone or in combination with wearable devices to collect real-time, objective measures of patient activity. In addition to their use as trial endpoints, PRO data may be used to support pharmaceutical labelling claims, clinical decision-making, clinical guideline development, and inform health policy.[6]

Historically, PROs have been assessed using paper-based questionnaires that have been evaluated psychometrically to establish their measurement properties. Increasingly PRO instruments are administered digitally as electronic patient-reported outcomes (ePROs). ePRO systems typically use a digital device such as a computer, tablet or smartphone to deliver the PRO instrument(s) to the patient.[7] Leveraging remote connections and notification capability, ePROs can be programmed to trigger alerts to the clinical/trials staff should a participant report the presence of one or more symptoms of clinical concern.[8, 9] In addition, ePROs offer the advantage of reducing data entry errors and respondent and administrative burden while facilitating data collection at specified time points and providing a record of the date and time entries are made.[7] Remote monitoring functionality enables trial participants to remain in their own homes at the same time as facilitating the surveillance of symptoms and disease status by the trial's clinical team.[8, 10]

PROs in trials of ATMPs

Due to their novelty, complexity and technical specificity, ATMPs bring 'new, unexplored risks to patients', including risks such as unwanted immunogenicity and severe toxicity.[11] PROs may be used to directly capture symptomatic adverse events (AEs) and the overall treatment burden from the patient perspective and, therefore, have potential to provide evidence on the safety and efficacy of an ATMP under investigation. In this way, PRO data can support post-treatment follow-up and risk management (including monitoring of the longer term effects on patients), provide essential data to inform real-world use, facilitate cost-benefit profiling, as well as support marketing authorisation.[2, 12] In addition, PROs have potential to support a fuller understanding of tolerability (the ability or desire of the patient to adhere to a specific dose or intensity of therapy) by providing direct measurements from the patient on how they are feeling and functioning while on treatment.[13, 14]. For patients, PROs in ATMP trials provide an opportunity to communicate outcomes of importance not captured by traditional clinical endpoints. PROs may also encourage patients to engage as participants, increase the likelihood of PRO claims in product labelling, and empower patients and clinicians to make more informed treatment decisions leading to better clinical outcomes.[15]

The PROmics ePRO system

The PROmics system is a bespoke, trial-specific electronic data capture system designed to collect and assess PRO data when patients receive an advanced cell therapy. PROmics captures PRO data relating to patient-reported adverse events and side effects, global tolerability of the medicinal product,

For peer review only - http://bmjopen.bmj.com/site/about/guidelines.xhtml

and quality of life and daily functioning tailored for each disease cohort. The PRO instruments selected for PROmics were identified in collaboration with patient partners and other stakeholders in a workshop held in 2018. The included PROs were selected to facilitate assessment of efficacy and tolerability by gathering preliminary evidence from the patient perspective on the benefits and risks of the ATMP. These data also have potential to inform the design and conduct of later-phase trials.[16]In addition to patient preference, selection was based upon regulatory recommendations, the psychometric properties of the PRO instruments, use in clinical practice and trials, and a need to ensure consistency in PRO reporting across disease cohorts (Table 1).

The following PRO instruments were selected for inclusion:

- The National Cancer Institute Patient-reported outcomes Version of the Common Terminology Criteria for Adverse Events (PRO-CTCAE) is a 78-item questionnaire that has been developed to characterise the frequency, severity and interference of symptomatic treatment toxicities from the patient perspective. The PRO-CTCAE was designed to be used as a companion to the CTCAE, the standard lexicon for AE reporting used in trials.[17-19]
- The FACT-G GP5 is a single item measuring patient-reported global treatment tolerability.[20]
- The EQ-5D-5L is a generic measure of self-rated health status. It consists of five items, each assessing a different health domain (mobility, self-care, usual activities, pain/discomfort, anxiety/depression), and a visual analogue scale (VAS) measuring global health status.[21]
- The FACIT-Fatigue scale is a 13-item generic PRO instrument measuring quality of life concerns relating to fatigue in chronic illness.[22, 23]
- The Fatigue Severity Scale (FSS) is a nine-item generic instrument that assesses the severity of fatigue and its impact on daily living.[24]
- The Primary Sclerosing Cholangitis Patient Reported Outcomes (PSC-PRO) instrument is a 42-item questionnaire measuring PSC symptoms and their impact.[25]

Table 1: PRO instruments included in the PROmics system for the POLARISE trial

PRO Instrument	Concept of interest	Disease cohort				Rationale for selection
		PSC	RA	LN	CD	
PRO-CTCAE	Burden (frequency, severity, interference) of symptomatic toxicities	X	X	X	X	Side effect and adverse event monitoring
FACT-G GP5	Global tolerability of treatment	X	X	X	X	Monitoring of treatment tolerability from the patient perspective
EuroQol 5D-5L (EQ5D-5L)	Health status	X	X	X	X	Local health reimbursement requirement
Fatigue Severity Scale (FSS)	Fatigue severity and impact on daily living	X	X	X	X	Preferred by PSC patients, use in previous trials, methodological consistency and to maximise comparability between trial arms
FACIT-Fatigue	Quality of life rating to fatigue	X	X	X	X	Recommended for use in RA patients by the European Medicines Association (EMA) and the International Consortium for Health Outcomes Measurement (ICHOM), methodological consistency, maximise

For peer review only - http://bmjopen.bmj.com/site/about/guidelines.xhtml

For peer review only

						comparability between trial arms
PSC-PRO	PSC symptom severity and impact	X				PSC-specific PRO instrument, patient preference, instrument developed with extensive patient input

CD = Crohn's Disease; LN = Lupus Nephritis; RA = Rheumatoid Arthritis; PSC = Primary Sclerosing Cholangitis

To complete the PRO instruments, patients access the PROmics system via an app on their personal tablet or smartphone, a data collection system known as "Bring Your Own Device (BYOD)".[7] The PROs are delivered in a standardised order for self-completion by the trial participant. Pre-programmed notifications will remind participants to submit their completed questionnaires, if they have not done so within a specified time-period. Clinical alerts, in the form of email notifications to the research site team, are triggered if a participant reports a high symptom burden. The alert, in the form of an email message to named trial staff at the relevant research site, prompts staff to login to the PROmics clinical dashboard, review the PRO scores for the corresponding trial ID number, and, if required, complete an adverse event (AE) report using the Common Terminology Criteria for Adverse Events (CTCAE).[26]

POLARISE Trial

The PROmics feasibility study is set in the context of the POLARISE Trial (EudraCT: 2019-003404-13; ISRCTN80103507), a single arm, multi-centre, phase II basket trial investigating the safety and activity of ORBCEL-C in the treatment of patients (N = 60) with immune-mediated inflammatory disease – specifically, Primary Sclerosing Cholangitis (PSC), Rheumatoid Arthritis (RA), Lupus Nephritis (LN) and Crohn's Disease (CD). These disease groups represent a highly heterogeneous sample in terms of age, physical impairment, and other disease-related characteristics. However, all have a shared pathogenesis and are associated with high symptom burden and reduced quality of life (QoL), necessitating the collection of PROs at scheduled time-points as well as notifications of patient-reported adverse events (AEs).[27] Remote collection of PRO data is possible through use of an ePRO system, with its specific mode of delivery and enhanced functionality, enabling these trial specific requirements to be met.

AIMS AND OBJECTIVES

To qualitatively assess the feasibility and acceptability of the PROmics system, an ePRO platform for use in trials of ATMPs. The planned study will evaluate the use of PROmics when deployed in a trial environment generally and, more specifically, in the POLARISE study, a phase II multi-disease ATMP trial in patients with immune-mediated inflammatory disease.

The specific study objectives are:

1. To explore with PROmics users, their' experiences of being trained with and using the PROmics ePRO system;
2. To explore decision-making with POLARISE trial participants who did not consent to report their PROs via PROmics or withdrew from using the PROmics;
3. To explore the benefits and shortcomings of PROmics and how these may be developed further;
4. To explore how the use of PROmics was managed by research staff and research participants in the context of the POLARISE trial.

METHODS AND ANALYSIS

For peer review only - http://bmjopen.bmj.com/site/about/guidelines.xhtml

This study will be reported in accordance with the Consolidated Criteria for Reporting Qualitative Research (COREQ).[28]

Design

A remote, qualitative, interview-based feasibility study (see figure 1) embedded within the POLARISE trial, a single arm, phase II ATMP basket trial recruiting from six National Health Service (NHS) trusts in the United Kingdom.

Participant selection

In total, it is estimated that 20 to 30 participants will be recruited to this feasibility study. Recruitment will proceed until data saturation, defined as the degree to which new data expresses what was expressed in previous data, is achieved.[29] Two participant groups, POLARISE trial participants (n = 10 – 15 participants) and members of the POLARISE research team (n = 10-15 participants) will be enrolled.

Sampling will be purposive for maximum variation for both POLARISE participants and research staff. Maximum variation where possible will be sought to ensure broad representation of disease groups, age, sex, ethnicity, research site/geographic location (POLARISE participants) and age, sex, clinical/research role, research site/geographic location (research staff).

To take part in the PROmics feasibility study, patients must be enrolled in the POLARISE trial (therefore meeting the trial eligibility criteria) and have either: 1) consented to report their PRO data (symptoms, tolerability, quality of life) using the PROmics ePRO system; 2) consented initially to report their PROs using the PROmics and subsequently withdrew from using the PROmics system to report their PRO data, reverting to paper questionnaires; or 3) declined to report their PROs using the PROmics software . Sampling will aim to recruit between three and five participants from each disease group and any POLARISE participants who decline to use or subsequently withdraw from using the PROmics system.

Participants who are research staff will be members of the research team who have been involved in the planning, delivery, administration and use of the PROmics ePRO system including: 1) research nurses working on the POLARISE trial; 2) POLARISE trial staff; and 3) staff involved in the management of POLARISE trial data. Research staff were enrolled to evaluate the feasibility, acceptability and ease of use of the clinician-facing components of the PROmics system (e.g., clinical dashboard) and to explore perceptions and attitudinal responses to ePRO data capture within an ATMP trial.

All PROmics participants must be able to converse in everyday English and be 18 years old or older. Exclusion criteria include being unable to read, understand, and complete questionnaires presented in English.

Recruitment and consent

All POLARISE patient participants will be eligible to take part in this feasibility study and will be provided with information packs relating to the feasibility study upon enrolment in the POLARISE trial. The first patient was registered to POLARISE on 19th April 2022 and the trial is estimated to recruit for two years. POLARISE participants will be involved in the trial for approximately 25 months. The estimated duration of the planned feasibility study is dependent on trial recruitment. Interviews will be scheduled with patient participants four to six months after enrolment into the POLARISE trial.

The PROmics feasibility study information packs will be tailored depending on whether a POLARISE participant has: 1) consented to report their PRO data via the PROmics system (accept); 2) consented to provide PRO data via PROmics and changed to paper-based PROs (withdraw); or 3) declined to report their PRO data via the PROmics software (decline). All versions (accept/withdraw/decline) of the information pack will include a cover letter, participant information sheet, and an expression of interest

For peer review only - http://bmjopen.bmj.com/site/about/guidelines.xhtml

form (+ reply paid envelope) to be returned by the participant to the PROmics research team if wish to take part in the feasibility study. POLARISE participants who decline to take part in the feasibility study (i.e., do not return an expression of interest form) will complete the PROs using their preferred method (i.e., using PROmics or completing paper forms) and will have no further contact with the PROmics research team.

POLARISE research team participants will be approached and provided with recruitment packs through liaison with the POLARISE trial sites. The POLARISE research staff recruitment packs will include a cover letter, participant information sheet, and expression of interest form.

A semi-structured interview conducted via telephone or videoconference (e.g., Zoom) will be arranged with all participants (patients and members of the POLARISE research team) who express an interest in participating in this feasibility study. Verbal consent will be sought at the time of the telephone/videoconference interview. A pre-specified consent script will enable consent to be taken via the telephone. Participants will be reminded of the study aims and that they are able to withdraw from the study at any time. The consenting process will be completed prior to commencing the interview and will be audio-recorded.

Data collection

Interviews will be arranged at a time point in the POLARISE trial to allow participants sufficient opportunity to use the PROmics system and to ensure accurate recall of their experiences whilst enrolled in the POLARISE trial (i.e., approximately 4-6 months after POLARISE trial enrolment). As the POLARISE trial is a national trial with multiple sites, this method of data collection was selected to enable: 1) ease of communication between participants and the PROmics research team and 2) ensure safe social distancing for COVID-19.

Semi-structured topic guides (see online supplemental file 1) will support patient and research staff interviews and will ensure key topics are consistently covered. The guides will include set/specific questions designed to elicit descriptions of participants' user experiences of the PROmics system, product perception, compliance, acceptability, and feasibility of use. The semi-structured nature of the planned interviews will enable the researcher conducting the interviews to explore topics in depth and accommodate emerging themes through prompts and questions that are informed by the participants' responses. All interviews will be audio-recorded using a digitally encrypted recorder and transcribed verbatim by a qualified, professional transcription service working within a confidentiality agreement. To confirm the accuracy of the interview transcripts, the transcripts will be reviewed against the audio-recording by the PROmics research team. All transcripts will be de-identified and a unique identifier will be assigned to each participant prior to undertaking data analyses. Data collection and analysis will take place concurrently and will continue until the research team agrees data saturation has been reached (per participant group and overall).

Data analysis

The de-identified interview transcripts will be entered into NVivo (Version 20) qualitative data analysis software and analysed using thematic analysis and the framework method described by Gale et al. to identify, describe, and report patterns in the data.[30-32] The framework method is considered appropriate for use with concrete data in studies where research outcomes are clearly defined.[32] Two researchers from the PROmics research team (CM, SEH) will independently code six interview transcripts (three per participant group). Coding will proceed inductively line-by-line to develop a set of initial codes. The researchers will compare codes to produce a consensus-based coding framework which will be checked by the wider research team before being applied across the data set. Codes will be grouped to form categories and categories refined to represent higher-level themes which encompass the full data set and present a clear, coherent and detailed interpretation of the data. Each participant group (i.e., patients and research staff) will be analysed separately and then compared later

For peer review only

For peer review only - http://bmjopen.bmj.com/site/about/guidelines.xhtml

in the analysis. The final analysis will be discussed with the POLARISE trial Patient and Public Involvement and Engagement (PPIE) group to establish the trustworthiness and validity of the findings.

Reflexivity

Reflexivity may be defined as researchers' acknowledgment of their own beliefs, judgements and practices and the role these play during the research process.[33] It is central to conducting a thematic analysis as the researcher plays an active role in the identification patterns within the data.[30, 31] The PROmics research team comprises experts in PROs (MC, SEH, CM), qualitative research (CM, SEH), clinical trials (AR, RM), ePROs (CF, EHD), and a consultant hepatologist (PN). It is nested within the larger POLARISE trial research team. Two experienced qualitative researchers (CM, SEH) will conduct the interviews and undertake data analysis. Reflexive research journals and memo-writing will be used to acknowledge the role of the researcher lens during the analytic process. None of the research team have any relationship with patient participants; however, it is possible that participants who are research staff at POLARISE trial sites may be known contacts to some members of the PROmics team.

PATIENT AND PUBLIC INVOLVEMENT

PPIE in the PROmics study and wider POLARISE trial was informed by the National Institute for Health Research (NIHR) guidance on the involvement of patient public contributors to research.[34] Patient partners were involved from the outset of the project. To date, PPIE members have informed the design and content of the PROmics ePRO system. A series of stakeholder meetings were convened to establish patient preferences for the format and layout of the PROmics system and to inform PRO selection. Prior to deployment in the POLARISE trial, usability testing of the PROmics system was completed with PPIE members. The outcomes of the stakeholder engagement events and usability testing are reported elsewhere. This protocol was reviewed by patient partner (RW) and the Liver and Gastro-Intestinal PPI Reference Group at the University of Birmingham and the Birmingham Rheumatology Research Patient Partnership (R2P2) who will also support the dissemination of information about the study. Patient and public involvement will be evaluated at the end of the project using the GRIPP-2 checklist.[35, 36]

ETHICS AND DISSEMINATION

Ethical approval for the PROmics feasibility study was granted as part of the POLARISE trial by London - West London & GTAC Research Ethics Committee (21/LO/0475). Local R&D approvals will be obtained prior to recruitment at sites. Informed consent will be obtained verbally and audio-recorded from all participants before data collection. Participants consent to being interviewed and the interviews being audio recorded and transcribed verbatim, and they acknowledge that their words can be used anonymously in the presentation of the research, that they can withdraw their data within seven working days of completing the interview, that participation is voluntary, and data management and storage is subject to the UK General Data Protection Regulation (2018). The results of this study will be disseminated via peer-reviewed journals, conference presentations and shared via social media. The PPIE groups and ATMP stakeholder networks will be consulted to maximise dissemination and impact.

DISCUSSION

Clinical trialists, regulators and policy-makers recognise increasingly the importance of the patient experience in the evaluation of the effectiveness, safety, and tolerability of medicinal products, including in trials of advanced cellular and gene therapies.[2, 37] Accordingly, PRO assessments are included in clinical trials to measure constructs such as symptom severity and functioning and health-related quality of life and, increasingly, these data are collected using ePROs. However, the impact on patients and

For peer review only - http://bmjopen.bmj.com/site/about/guidelines.xhtml

the feasibility and acceptability of ePRO data capture has yet to be established fully in ATMP trials. This qualitative study aims to provide a deeper understanding into the use ePROs in clinical trials of advanced therapies. Barriers and enablers to the use of PROmics, a new ePRO data capture system, will be explored within an ATMP trial for patients with immune-mediated inflammatory disease. These qualitative data will be supplemented with further quantitative findings at the end of the POLARISE trial including compliance and rates of missing data. The findings will inform recommendations to improve the PROmics system, to enhance PRO strategy in future studies; promote efficient use of measures, data completeness, and acceptability to trial participants; inform future analyses and sample size estimation; and support future implementation of ePROs in ATMP trials and routine care.

The study will incorporate the views of a range of stakeholders. The involvement of trial participants from different disease cohorts and from different geographical locations in the UK will enhance variation in the study sample. These patient participants will provide insights into user confidence with digital technology and the completion of PROs electronically, time points of administration, and the overall user experience. Inclusion of trial participants who declined or withdrew from using the PROmics will allow us to explore potential barriers and solutions to participation including inclusion of underserved populations. The views of trial staff and site research nurses will help understand PROmics integration with local IT services and the administrative burden on staff as well as barriers to and enablers of the use of PROmics for data collection, trial participant surveillance, and AE reporting.

Strengths and limitations of this study

The use of qualitative methods is a strength of this study, allowing in-depth exploration of user experience. Nesting the feasibility study within the POLARISE trial will enable timely data collection thereby limiting recall bias. The study sample will include research participants from NHS hospitals from different regions of the UK which may enhance the transferability and generalisability of the study findings. However, as not all trial participants will take part in the feasibility study, sampling is potentially biased. Digital exclusion, particularly for underserved populations that may have limited access to digital devices, contributes a further source of bias to the proposed study.[38] Sampling bias will be addressed by recruiting purposively for maximum variation and through the inclusion of participants who declined to use the PROmics system or who reverted to the use of paper-based PROs at some point in the trial. Questionnaires programmed onto the PROmics system are currently in UK English, however, if the solution proves feasible, inclusion of translated, culturally validated measures will be undertaken in future work.

Conclusions

The number of ATMP trials continues to grow year-on-year, offering potentially powerful new treatments for patients with rare or life-threatening diseases.[39] As with all investigational medicinal products, demonstrating efficacy, safety and tolerability of new ATMPs is crucial. Electronic PROs have the potential to contribute important evidence of efficacy, safety and tolerability (both in the short and longer term) for these novel therapies and to support patient monitoring and risk management. Data collected via the PROmics system has the potential to provide new insights into the reporting of symptomatic AEs specific to the advanced therapies context whilst evaluation of the feasibility and acceptability of the PROmics system will contribute new knowledge to support the development of ePRO reporting formats that meet the unique needs of advanced therapies, for the benefit of all who receive these novel treatments.

ACKNOWLEDGMENTS

The authors thank POLARISE Trial Steering Committee, POLARISE Scientific Advisory Group, PROmics Project Management Group, and Patient and Public Involvement (PPI) group members for their input and support of the project.

For peer review only - http://bmjopen.bmj.com/site/about/guidelines.xhtml

Contributors

The feasibility study concept and design were conceived by AR, AmR, PN, MC, RW, RH, GP. CB, AR will support recruitment. EHD, CF, GM were responsible for the development and programming of PROmics in Atom5™. EHD, CF will support POLARISE data collection using the PROmics system. SEH and CM will consent participants and undertake the interviews and data analysis. The protocol was written by AmR, RM, MC, AR, RW, PN. SEH prepared the first draft of the manuscript. AW will provide administrative support to the project. All authors (SEH, CM AR, AmR, RM, CB, EHD, CF, GM, RH, GP, RW, PN, MC) reviewed the manuscript, provided edits and approved the final version.

Funding

The author(s) disclosed receipt of the following financial support for the research, authorship, and/or publication of this article: This work was supported by the National Institute for Health Research (NIHR) Birmingham Biomedical Research Centre, Midlands-Wales Advanced Therapies Treatment Centre (MW-ATTC) programme grant from Innovate UK to a consortium of partners including Health Technology Wales, the Welsh Blood Service and the University of Birmingham (Grant number: IUK: 104232) and Innovate UK (part of UK Research and Innovation) grant Patient-reported outcomes assessment to support accelerated access to advanced cell and gene therapies: PROmics (Grant No: IUK: 104777). The funder was not involved in any aspect of the research work.

Competing Interests

SEH receives funding from the receives funding from the NIHR Oxford-Birmingham Blood and Transplant Research Unit (BTRU) in Precision Therapeutics, UK Research and Innovation (UKRI) and declares personal fees from Cochlear Ltd, Astra Zeneca, and Aparito Ltd. *MC* is a National Institute for Health Research (NIHR) Senior Investigator and receives funding from the National Institute for Health Research (NIHR) Birmingham Biomedical Research Centre, the NIHR Oxford-Birmingham Blood and Transplant Research Unit (BTRU) in Precision Therapeutics, the NIHR Surgical Reconstruction and Microbiology Research Centre and NIHR ARC West Midlands at the University of Birmingham and University Hospitals Birmingham NHS Foundation Trust, Health Data Research UK, UKRI, Macmillan Cancer Support, UCB Pharma Gilead, Janssen and GSK. MC has received personal fees from Astellas, Aparito Ltd, CIS Oncology, Takeda, Merck, Daiichi Sankyo, Glaukos, GSK and the Patient-Centered Outcomes Research Institute (PCORI) outside the submitted work. *CM* receives funding from NIHR Surgical Reconstruction and Microbiology Research Centre (SRMRC), the NIHR Oxford-Birmingham Blood and Transplant Research Unit (BTRU) in Precision Therapeutics UKRI, and declares personal fees from Aparito Ltd outside the submitted work. All other authors have no interests to declare.

Disclaimer

The views expressed are those of the authors and not necessarily those of Innovate UK.

Patient consent for publication

Not required.

Provenance and peer review

Not commissioned, externally peer reviewed.

Open access

This is an open access article distributed in accordance with the Creative Commons Attribution 4.0 International license (CC BY 4.0), which permits others to distribute, remix, adapt, build upon this work, and license their derivative works on different terms, provided the original work is properly cited, appropriate credit is given, and any changes made indicated. See: http:// https://creativecommons.org/licenses/by/4.0/.

REFERENCES

1. Elverum K, Whitman M. Delivering cellular and gene therapies to patients: solutions for realizing the potential of the next generation of medicine. *Gene Ther* 2020;27:537-44. doi: 10.1038/s41434-019-0074-7

2. Lasiter L, Campbell A, Basch E, et al. Use of patient-reported outcomes to understand & measure the patient experience of novel cell and gene therapies. *Ther Innov Regul Sci* 2020;54:1566-75. doi: 10.1007/s43441-020-00184-6

For peer review only

For peer review only - http://bmjopen.bmj.com/site/about/guidelines.xhtml

3. Hollingsworth I. Increasing patient access to advanced therapies. *International Biopharmaceutical Industry* 2020; 3(1). https://ww https://www.theattcnetwork.co.uk/news/increasing-patient-access-to-advanced-therapies-the-uk-perspective.
4. Kyte D, Ives J, Draper H, et al. Current practices in patient-reported outcome (PRO) data collection in clinical trials: a cross-sectional survey of UK trial staff and management. *BMJ Open* 2016;6:e012281. doi: 10.1136/bmjopen-2016-012281
5. FDA. Guidance for industry: Patient-reported outcome measures: use in medicinal product development to support labelling claims, 2009. https://www.fda.gov/regulatory-information/search-fda-guidance-documents/patient-reported-outcome-measures-use-medical-product-development-support-labeling-claims 6. Calvert M, Kyte D, Mercieca-Bebber R, et al. Guidelines for inclusion of patient-reported outcomes in clinical trial protocols: the SPIRIT-PRO Extension. *JAMA* 2018;319:483-94. doi: 10.1001/jama.2017.21903
7. Coons SJ, Eremenco S, Lundy JJ, et al. Capturing patient-reported outcome (pro) data electronically: the past, present, and promise of epro measurement in clinical trials. *Patient* 2015;8:301-09. doi: 10.1007/s40271-014-0090-z
8. Holch P, Pini S, Henry AM, et al. eRAPID electronic patient self-reporting of adverse-events: Patient Information and advice: a pilot study protocol in pelvic radiotherapy. *Pilot Feasibility Stud* 2018;4:110. doi: 10.1186/s40814-018-0304-6
9. Kyte D, Ives J, Draper H, et al. Management of patient-reported outcome (pro) alerts in clinical trials: a cross sectional survey. *PLoS One* 2016;11:e0144658-e58. doi: 10.1371/journal.pone.0144658
10. Basch E, Deal AM, Kris MG, et al. Symptom monitoring with patient-reported outcomes during routine cancer treatment: a randomized controlled trial. *J Clin Oncol* 2016;34:557-65. doi: 10.1200/JCO.2015.63.0830
11. European Medinces Agency. Guideline on Safety and Efficacy Follow-Up - Risk Management of Advanced Therapy Medicinal Products. 2008. https://www.ema.europa.eu/en/documents/scientific-guideline/guideline-safety-efficacy-follow-risk-management-advanced-therapy-medicinal-products_en.pdf.
12. Calvert M, Kyte D, Price G, et al. Maximising the impact of patient reported outcome assessment for patients and society. *BMJ* 2019;364:k5267. doi: 10.1136/bmj.k5267
13. Kluetz PG, Kanapuru B, Lemery S, et al. Informing the Tolerability of Cancer Treatments Using Patient-Reported Outcome Measures: Summary of an FDA and Critical Path Institute Workshop. *Value Health* 2018;21:742-47. doi: https://doi.org/10.1016/j.jval.2017.09.009
14. European Medicines Agency. Appendix 2 to the guideline on the evaluation of anticancer medicinal products in man: The use of patient-reported outcome (PRO) measures in oncology studies. 2016. https://www.ema.europa.eu/en/documents/other/appendix-2-guideline-evaluation-anticancer-medicinal-products-man_en.pdf.
15. Coleman RL, Beck JT, Baranda JC, et al. The use of patient-reported outcome measures in phase i oncology clinical trials. *OCL* 2021;99(7):444-53. doi: 10.1159/000514874
16. Retzer A, Aiyegbusi OL, Rowe A, et al. The value of patient-reported outcomes in early-phase clinical trials. *Nat Med* 2022;28:18-20. doi: 10.1038/s41591-021-01648-4
17. Smith AW, Mitchell SA, K. De Aguiar C, et al. News from the NIH: Person-centered outcomes measurement: NIH-supported measurement systems to evaluate self-assessed health, functional performance, and symptomatic toxicity. *Transl Behav Med* 2016;6:470-74. doi: 10.1007/s13142-015-0345-9
18. Kluetz PG, Chingos DT, Basch EM, et al. Patient-reported outcomes in cancer clinical trials: measuring symptomatic adverse events with the national cancer institute's patient-reported outcomes version of the common terminology criteria for adverse events (PRO-CTCAE). *Am Soc Clin Oncol Educ Book* 2016;36:67-73. doi: 10.1200/EDBK_159514
19. Dueck AC, Mendoza TR, Mitchell SA, et al. Validity and reliability of the us national cancer institute's patient-reported outcomes version of the common terminology criteria for adverse events (PRO-CTCAE). *JAMA Oncol* 2015;1:1051-59. doi: 10.1001/jamaoncol.2015.2639
20. Pearman TP, Beaumont JL, Mroczek D, et al. Validity and usefulness of a single-item measure of patient-reported bother from side effects of cancer therapy. *Cancer* 2018;124:991-97. doi: https://doi.org/10.1002/cncr.31133
21. Herdman M, Gudex C, Lloyd A, et al. Development and preliminary testing of the new five-level version of EQ-5D (EQ-5D-5L). *Qual Life Res* 2011;20:1727-36. doi: 10.1007/s11136-011-9903-x

For peer review only - http://bmjopen.bmj.com/site/about/guidelines.xhtml

22. Cella DF, Tulsky DS, Gray G, et al. The Functional Assessment of Cancer Therapy scale: development and validation of the general measure. *J Clin Oncol* 1993;11:570-79. doi: 10.1200/jco.1993.11.3.570
23. Webster K, Cella D, Yost K. The Functional Assessment of Chronic Illness Therapy (FACIT) Measurement System: properties, applications, and interpretation. *Health Quality Life* 2003;1:79. doi: 10.1186/1477-7525-1-79
24. Krupp LB, LaRocca NG, Muir-Nash J, et al. The Fatigue Severity Scale: application to patients with multiple sclerosis and systemic lupus erythematosus. *Arch Neurol* 1989;46:1121-23. doi: 10.1001/archneur.1989.00520460115022
25. Younossi ZM, Afendy A, Stepanova M, et al. Development and validation of a primary sclerosing cholangitis–specific patient-reported outcomes instrument: the PSC PRO. *Hepatology* 2018;68:155-65. doi: https://doi.org/10.1002/hep.29664
26. CTE Programe. Common Terminology Criteria for Adverse Events (CTCAE) v4.0: NCI; [Available from: https://evs.nci.nih.gov/ftp1/CTCAE/CTCAE_4.03/CTCAE_4.03_2010-06-14_QuickReference_5x7.pdf.
27. David T, Ling SF, Barton A. Genetics of immune-mediated inflammatory diseases. *Clin Exp Immunol* 2018;193:3-12. doi: 10.1111/cei.13101
28. Tong A, Sainsbury P, Craig J. Consolidated criteria for reporting qualitative research (COREQ): a 32-item checklist for interviews and focus groups. *Int J Qual Health Care* 2007;19:349-57. doi: 10.1093/intqhc/mzm042
29. Saunders B, Sim J, Kingstone T, et al. Saturation in qualitative research: exploring its conceptualization and operationalization. *Qual Quant* 2018;52:1893-907. doi: 10.1007/s11135-017-0574-8
30. Braun V, Clarke V. Using thematic analysis in psychology. *Qual Res Psychol* 2006;3:77-101. doi: 10.1191/1478088706qp063oa
31. Braun V, Clarke V. Reflecting on reflexive thematic analysis. *Quali Res Sport, Exerc Health* 2019;11:589-97. doi: 10.1080/2159676X.2019.1628806
32. Gale NK, Heath G, Cameron E, et al. Using the framework method for the analysis of qualitative data in multi-disciplinary health research. *BMC Med Res Methodol* 2013;13:117. doi: 10.1186/1471-2288-13-117
33. Finlay L. Reflexivity: An essential component for all research? *Br J Occup Ther* 1998;61:453-56. doi: 10.1177/030802269806101005
34. National Institute for Health Research. Centre for Dissemination and Engagement recognition payments for public contributors 2020. Available from: https://www.nihr.ac.uk/documents/centre-for-engagement-and-dissemination-recognition-payments-for-public-contributors/24979.
35. Staniszewska S, Brett J, Simera I, et al. GRIPP2 reporting checklists: tools to improve reporting of patient and public involvement in research. *Res Involv Engagem* 2017;3:13. doi: 10.1186/s40900-017-0062-2
36. Kok M, Gibson A, Evans D, et al. Guidance document: evaluating public involvement in research. 2018. http://www.phwe.org.uk/wp-content/uploads/Guidance-on-evaluating-Public-Involvement-in-research.pdf.
37. Chakraborty R, Sidana S, Shah GL, et al. Patient-reported outcomes with chimeric antigen receptor t cell therapy: challenges and opportunities. *Biol Blood Marrow Transplant* 2019;25:e155-e62. doi: https://doi.org/10.1016/j.bbmt.2018.11.025
38. Calvert MJ, Cruz Rivera S, Retzer A, et al. Patient reported outcome assessment must be inclusive and equitable. *Nat Med* 2022;28:1120-24. doi: 10.1038/s41591-022-01781-8
39. Hanna E, Rémuzat C, Auquier P, et al. Advanced therapy medicinal products: current and future perspectives. *J Mark Access Health Policy* 2016;4:10.3402/jmahp.v4.31036. doi: 10.3402/jmahp.v4.31036

FIGURE LEGEND

Figure 1. PROmics qualitative feasibility study embedded within the POLARISE trial. Flow chart of participants.

For peer review only - http://bmjopen.bmj.com/site/about/guidelines.xhtml

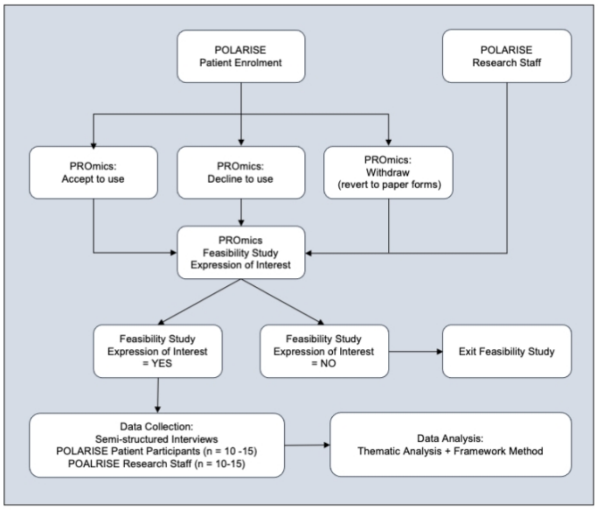

Figure 1. PROmics qualitative feasibility study embedded within the POLARISE trial. Flow chart of participants.

107x60mm (300 x 300 DPI)

SUPPLEMENTAL FILE 1 – INTERVIEW TOPIC GUIDE

PATIENTS

1. Restate the purpose of the interview: *Discuss PROmics system*
2. Select the interview structure based on patient's ACCEPT, WITHDRAW, DECLINE status: If ACCEPT, ask ACCEPT questions only. If WITHDRAW, ask ACCEPT and WITHDRAW questions. If DECLINE, ask DECLINE questions only.
3. Any questions or clarification before beginning? Feel free to take a break at any point during the interview.
4. Materials: paper questionnaires, app with PROmics software for reference

ACCEPT

1. Why did you choose to use the PROmics software?
- Was there anything that made you question if you should use it? (FURTHER PROBES: Burden, confidence with technology)
- Were you given enough information?
2. Did you find the initial training with the PROmics software helpful?
- Was there anything particularly good or bad about the training?
- How could it be improved?
3. How did you find using the PROmics software?
- Did you feel like you had enough support?
- How could the support for patients using the PROmics software be improved?
4. How did use of the PROmics software fit in with your other trial-related activities? (FURTHER PROBE: Burden)
- Did it make you feel more or less engaged with the trial overall? How?
- Did it make you feel like you were getting better or worse care during the trial? How?
5. How did you find the PROmics software to use?
- What did you like? Why?
- What didn't you like? Why?
- How could it be improved?
- How does it compare to paper questionnaires? (SHOW: Paper questionnaires)
6. Would you recommend for others to use the PROmics software if they participating in a trial? Why?

WITHDRAW

1. Why did you decide to stop using the PROmics software?
- What could have been done to help you continue to use the software?

DECLINE

1. Why did you choose not to use the PROmics software?

For peer review only - http://bmjopen.bmj.com/site/about/guidelines.xhtml

UNIVERSITY OF BIRMINGHAM | CPROR MAKING PATIENT CENTRED CARE A REALITY

- Was there anything that made you question if you should use it? (FURTHER PROBES: Burden, confidence with technology)
- Were you given enough information?

2. How does the PROmics software compare to paper questionnaires? (SHOW: app)

- Do you think you would use the PROmics software if you were in a trial again?

RESEARCH TEAM

1. Restate the purpose of the interview: *Discuss PROmics system*
2. Select the interview structure based on participant's experience: Site Research Nurse/ other. If OTHER, ask OTHER questions only. If SITE RESEARCH NURSE, ask SITE RESEARCH NURSE and OTHER questions.
3. Any questions or clarification before beginning? Feel free to take a break at any point during the interview.
4. Materials: paper questionnaires, demo iPad with PROmics software for reference

SITE RESEARCH NURSE

1. Did you find the initial training with the PROmics software helpful?

- Was there anything particularly good or bad about the training?
- How could it be improved?
- Were you given enough information?

2. How did you find using the PROmics software?

- Did you feel like you had enough support?
- How could the support for staff using the PROmics software be improved?

3. How did you find the PROmics software to use?

- What did you like? Why?
- What didn't you like? Why?
- How could it be improved?
- How does it compare to paper questionnaires? (SHOW: Paper questionnaires)

4. How did using the PROmics software impact on your care of patients?

- Was it better/worse?
- How could this be improved?

5. Did you feel equipped to train patients to use the PROmics software?

- How could this be improved?

6. Did you feel equipped to support patients to use the PROmics software during the trial?

- How could this be improved?

OTHER

1. How did use of the PROmics software fit in with your other trial-related activities? (FURTHER PROBES: Burden, workload)

- Was there anything that made you question if it should be used in the trial? (FURTHER PROBES: Burden, confidence with technology)
- Would you recommend that the PROmics software is used in future trials?

For peer review only - http://bmjopen.bmj.com/site/about/guidelines.xhtml

For peer review only

BMJ Open

Feasibility of a new electronic patient-reported outcome (ePRO) system for an advanced therapy clinical trial in immune-mediated inflammatory disease (PROmics): protocol for a qualitative feasibility study

Journal:	*BMJ Open*
Manuscript ID	bmjopen-2022-063199.R2
Article Type:	Protocol
Date Submitted by the Author:	11-Aug-2022
Complete List of Authors:	Hughes, Sarah; University of Birmingham, Centre for Patient Reported Outcome Research, Institute of Applied Health Research<br>McMullan, Christel; University of Birmingham, Centre for Patient Reported Outcome Research (CPROR), Institute of Applied Health Research<br>Rowe, Anna; University of Birmingham, National Institute for Health Research (NIHR) Biomedical Research Centre; University of Birmingham, Cancer Research UL Clinical Trials Unit<br>Retzer, Ameeta; University of Birmingham, Centre for Patient Reported Outcome Research<br>Malpass, Rebecca; University of Birmingham, Biomedical Research Centre; University of Birmingham, Cancer Research UK Clinical Trials Unit<br>Bathurst, Camilla; University of Birmingham, National Institute for Health Research (NIHR) Biomedical Research Centre; University of Birmingham, Cancer Research UK Clinical Trials Unit<br>Davies, Elin Haf; Aparito<br>Frost, Chris; Aparito<br>McNamara, Gary; Aparito<br>Harding, Rosie; University of Birmingham, Birmingham Law School<br>Price, Gary; University of Birmingham, Patient Partner, Centre for Patient Reported Outcome Research (CPROR)<br>Wilson, Roger; NCRI Consumer Forum, Sarcoma Patients Euronet<br>Walker, Anita; University of Birmingham, Centre for Patient Reported Outcome Research, Institute of Applied Health Research<br>Newsome, Philip; University of Birmingham, Institute of Immunology and Immunotherapy<br>Calvert , Melanie ; University of Birmingham, Centre for Patient Reported Outcomes Research and Institute of Applied Health Research
**Primary Subject Heading**:	Immunology (including allergy)
Secondary Subject Heading:	Health services research
Keywords:	IMMUNOLOGY, Information technology < BIOTECHNOLOGY & BIOINFORMATICS, RHEUMATOLOGY, Hepatology < INTERNAL MEDICINE, Inflammatory bowel disease < GASTROENTEROLOGY

Feasibility of a new electronic patient-reported outcome (ePRO) system for an advanced therapy clinical trial in immune-mediated inflammatory disease (PROmics): protocol for a qualitative feasibility study

Sarah E Hughes,[1-3,15] Christel McMullan,[1,4,5,15] Anna Rowe,[6,7] Ameeta Retzer,[1,3,4] Rebecca Malpass,[6,7] Camilla Bathurst,[6,7] Elin Haf Davies,[8] Chris Frost,[8] Gary McNamara,[9] Rosie Harding,[10] Gary Price,[1] Roger Wilson,[11] Anita Walker,[1] Philip Newsome,[1,6,12,13] Melanie Calvert[1-4,6,13,14,15]

[1]Centre for Patient Reported Outcome Research (CPROR), Institute of Applied Health Research, University of Birmingham, Birmingham, UK
[2]National Institute for Health Research (NIHR) Applied Research Centre (ARC) West Midlands, Birmingham, UK
[3]Birmingham Health Partners Centre for Regulatory Science and Innovation, University of Birmingham, Birmingham, UK
[4]National Institute for Health Research (NIHR) Surgical Reconstruction and Microbiology Research Centre, University of Birmingham, Birmingham, UK
[5]Centre for Trauma Science Research, University of Birmingham, Birmingham, UK
[6]National Institute for Health Research (NIHR) Birmingham Biomedical Research Centre
[7]Cancer Research UK Clinical Trials Unit, University of Birmingham, Birmingham, UK
[8]Aparito Limited, Wrexham, UK
[9]Cognitant, UK
[10]Birmingham Law School, University of Birmingham, UK
[11]Patient Partner, Member of the National Cancer Research Institute (NCRI) Consumer Form, UK
[12]Institute of Immunology and Immunotherapy, University of Birmingham, Birmingham, UK
[13]Midlands Health Data Research UK, Birmingham, UK
[14]DEMAND Hub, University of Birmingham, Birmingham, UK
[15]NIHR Oxford-Birmingham Blood and Transplant Research Unit (BTRU) in Precision Therapeutics

Correspondence to:
Sarah Hughes
Centre for Patient Reported Outcome Research
Institute for Applied Health Research
University of Birmingham
Edgbaston
B15 2TT
s.e.hughes@bham.ac.uk
ORCID: 0000-0001-5656-1198

Word count: 4082 words (Excluding tables, acknowledgements, and references)

For peer review only

For peer review only - http://bmjopen.bmj.com/site/about/guidelines.xhtml

ABSTRACT

Introduction The use of electronic patient-report outcome (ePRO) systems to capture PRO data in clinical trials is increasing; however, their feasibility, acceptability, and utility in clinical trials of advanced therapy medicinal products (ATMPs) are not yet well understood. This protocol describes a qualitative study that aims to evaluate the feasibility and acceptability of ePRO data capture using a bespoke, trial-specific ePRO system (the PROmics system) within an advanced therapy trial involving patients with immune-mediated inflammatory disease (Rheumatoid Arthritis, Lupus, Primary Sclerosing Cholangitis and Crohn's Disease).

Methods and analysis This protocol is for a remote, qualitative, interview-based feasibility study embedded within the POLARISE trial, a single arm, phase II, multi-site ATMP basket trial in the United Kingdom. 10-15 patients enrolled in the POLARISE trial and 10-15 research team members at the ATMP trial sites will be recruited. Participants will take part in semi-structured interviews which will be transcribed verbatim and analysed thematically according to the framework method. Data collection and analysis will occur concurrently and iteratively. Researcher triangulation will be used to achieve a consensus-based analysis, enhancing rigor and trustworthiness.

Ethics and dissemination This study was approved by the London - West London & GTAC Research Ethics Committee (Ref: 21/LO/0475). Informed consent will be obtained from all participants prior to data collection. The study findings will be published in peer-review journals and disseminated via conference presentations and other media. Our patient and public involvement and engagement (PPIE) group and ATMP stakeholder networks will be consulted to maximise dissemination and impact.

Trial registration number POLARISE trial: EudraCT 2019-003404-13, ISRCTN80103507.

For peer review only

For peer review only - http://bmjopen.bmj.com/site/about/guidelines.xhtml

STRENGTHS AND LIMITATIONS OF THIS STUDY

- The use of an ePRO system to collect patient-reported outcome (PRO) data as part of an early-phase ATMP (advanced therapy medicinal product) clinical trial with patients with immune-mediated inflammatory disease could help to identify symptom burden and adverse side effects in research participants earlier, enhancing patient safety.
- This study will provide data on the feasibility and acceptability of the PROmics approach to enhance future PRO strategy in later phases, promoting efficient use of measures; data completeness; acceptability to trial participants; and inform future analyses and sample size estimation.
- The study sample will include research participants from National Health Service (NHS) hospitals from different regions of the United Kingdom (UK) which may enhance the transferability and generalisability of the study findings.
- The study sample will include only those trial participants who consent to take part in this electronic PRO (ePRO) feasibility sub-study and therefore recruitment may be subject to selection bias.

For peer review only

For peer review only - http://bmjopen.bmj.com/site/about/guidelines.xhtml

INTRODUCTION

The use of stem cells, gene therapy and tissue engineering in the treatment of disease or injury represents an emerging and rapidly developing approach to medicine.[1, 2] These advanced therapy medicinal products (ATMPs) have the potential to transform the treatment of some diseases (e.g., sickle cell disease, cancer, haemophilia), removing the need for long-term medical care. In the United Kingdom (UK) there has been steady year-on-year growth in the number of clinical trials of ATMPs.[3]

Endpoints in clinical trials are designed to assess the effects of an intervention, including the efficacy, safety and tolerability of the medicinal product. As an adjunct to clinical endpoint data, patient-reported outcome (PRO) data, when captured in a rigorous and scientific manner, can provide further, valuable evidence of treatment benefit or risk.[4, 5] A PRO is "any report of the status of a patient's health condition that comes directly from the patient, without interpretation of the patient's response by a clinician or anyone else".[5] PRO data include patient perspectives of symptom burden, functioning, and their health-related quality of life and are typically captured using validated, self-report questionnaires. PROs may be used alone or in combination with wearable devices to collect real-time, objective measures of patient activity. In addition to their use as trial endpoints, PRO data may be used to support pharmaceutical labelling claims, clinical decision-making, clinical guideline development, and inform health policy.[6]

Historically, PROs have been assessed using paper-based questionnaires that have been evaluated psychometrically to establish their measurement properties. Increasingly PRO instruments are administered digitally as electronic patient-reported outcomes (ePROs). ePRO systems typically use a digital device such as a computer, tablet or smartphone to deliver the PRO instrument(s) to the patient.[7] Leveraging remote connections and notification capability, ePROs can be programmed to trigger alerts to the clinical/trials staff should a participant report the presence of one or more symptoms of clinical concern.[8, 9] In addition, ePROs offer the advantage of reducing data entry errors and respondent and administrative burden while facilitating data collection at specified time points and providing a record of the date and time entries are made.[7] Remote monitoring functionality enables trial participants to remain in their own homes at the same time as facilitating the surveillance of symptoms and disease status by the trial's clinical team.[8, 10]

PROs in trials of ATMPs

Due to their novelty, complexity and technical specificity, ATMPs bring 'new, unexplored risks to patients', including risks such as unwanted immunogenicity and severe toxicity.[11] PROs may be used to directly capture symptomatic adverse events (AEs) and the overall treatment burden from the patient perspective and, therefore, have potential to provide evidence on the safety and efficacy of an ATMP under investigation. In this way, PRO data can support post-treatment follow-up and risk management (including monitoring of the longer term effects on patients), provide essential data to inform real-world use, facilitate cost-benefit profiling, as well as support marketing authorisation.[2, 12] In addition, PROs have potential to support a fuller understanding of tolerability (the ability or desire of the patient to adhere to a specific dose or intensity of therapy) by providing direct measurements from the patient on how they are feeling and functioning while on treatment.[13, 14]. For patients, PROs in ATMP trials provide an opportunity to communicate outcomes of importance not captured by traditional clinical endpoints. PROs may also encourage patients to engage as participants, increase the likelihood of PRO claims in product labelling, and empower patients and clinicians to make more informed treatment decisions leading to better clinical outcomes.[15]

The PROmics ePRO system

The PROmics system is a bespoke, trial-specific electronic data capture system designed to collect and assess PRO data when patients receive an advanced cell therapy. PROmics captures PRO data relating to patient-reported adverse events and side effects, global tolerability of the medicinal product,

For peer review only - http://bmjopen.bmj.com/site/about/guidelines.xhtml

and quality of life and daily functioning tailored for each disease cohort. The PRO instruments selected for PROmics were identified in collaboration with patient partners and other stakeholders in a workshop held in 2018. The included PROs were selected to facilitate assessment of efficacy and tolerability by gathering preliminary evidence from the patient perspective on the benefits and risks of the ATMP. These data also have potential to inform the design and conduct of later-phase trials.[16]In addition to patient preference, selection was based upon regulatory recommendations, the psychometric properties of the PRO instruments, use in clinical practice and trials, and a need to ensure consistency in PRO reporting across disease cohorts (Table 1).

The following PRO instruments were selected for inclusion:

- The National Cancer Institute Patient-reported outcomes Version of the Common Terminology Criteria for Adverse Events (PRO-CTCAE) is a 78-item questionnaire that has been developed to characterise the frequency, severity and interference of symptomatic treatment toxicities from the patient perspective. The PRO-CTCAE was designed to be used as a companion to the CTCAE, the standard lexicon for AE reporting used in trials.[17-19]
- The FACT-G GP5 is a single item measuring patient-reported global treatment tolerability.[20]
- The EQ-5D-5L is a generic measure of self-rated health status. It consists of five items, each assessing a different health domain (mobility, self-care, usual activities, pain/discomfort, anxiety/depression), and a visual analogue scale (VAS) measuring global health status.[21]
- The FACIT-Fatigue scale is a 13-item generic PRO instrument measuring quality of life concerns relating to fatigue in chronic illness.[22, 23]
- The Fatigue Severity Scale (FSS) is a nine-item generic instrument that assesses the severity of fatigue and its impact on daily living.[24]
- The Primary Sclerosing Cholangitis Patient Reported Outcomes (PSC-PRO) instrument is a 42-item questionnaire measuring PSC symptoms and their impact.[25]

Table 1. PRO instruments included in the PROmics system for the POLARISE trial

PRO Instrument	Concept of interest	Disease cohort				Rationale for selection
		PSC	RA	LN	CD	
PRO-CTCAE	Burden (frequency, severity, interference) of symptomatic toxicities	X	X	X	X	Side effect and adverse event monitoring
FACT-G GP5	Global tolerability of treatment	X	X	X	X	Monitoring of treatment tolerability from the patient perspective
EuroQol 5D-5L (EQ5D-5L)	Health status	X	X	X	X	Local health reimbursement requirement
Fatigue Severity Scale (FSS)	Fatigue severity and impact on daily living	X	X	X	X	Preferred by PSC patients, use in previous trials, methodological consistency and to maximise comparability between trial arms
FACIT-Fatigue	Quality of life rating to fatigue	X	X	X	X	Recommended for use in RA patients by the European Medicines Association (EMA) and the International Consortium for Health Outcomes Measurement

For peer review only - http://bmjopen.bmj.com/site/about/guidelines.xhtml

						(ICHOM), methodological consistency, maximise comparability between trial arms
PSC-PRO	PSC symptom severity and impact	X				PSC-specific PRO instrument, patient preference, instrument developed with extensive patient input

CD = Crohn's Disease; LN = Lupus Nephritis; RA = Rheumatoid Arthritis; PSC = Primary Sclerosing Cholangitis.

To complete the PRO instruments, patients access the PROmics system via an app on their personal tablet or smartphone, a data collection system known as "Bring Your Own Device (BYOD)".[7] The PROs are delivered in a standardised order for self-completion by the trial participant. Pre-programmed notifications will remind participants to submit their completed questionnaires, if they have not done so within a specified time-period. Clinical alerts, in the form of email notifications to the research site team, are triggered if a participant reports a high symptom burden. The alert, in the form of an email message to named trial staff at the relevant research site, prompts staff to login to the PROmics clinical dashboard, review the PRO scores for the corresponding trial ID number, and, if required, complete an adverse event (AE) report using the Common Terminology Criteria for Adverse Events (CTCAE).[26]

POLARISE trial

The PROmics feasibility study is set in the context of the POLARISE trial (EudraCT: 2019-003404-13; ISRCTN80103507), a single arm, multi-centre, phase II basket trial investigating the safety and activity of ORBCEL-C in the treatment of patients (N = 60) with immune-mediated inflammatory disease – specifically, Primary Sclerosing Cholangitis (PSC), Rheumatoid Arthritis (RA), Lupus Nephritis (LN) and Crohn's Disease (CD). These disease groups represent a highly heterogeneous sample in terms of age, physical impairment, and other disease-related characteristics. However, all have a shared pathogenesis and are associated with high symptom burden and reduced quality of life (QoL), necessitating the collection of PROs at scheduled time-points as well as notifications of patient-reported adverse events (AEs).[27] Remote collection of PRO data is possible through use of an ePRO system, with its specific mode of delivery and enhanced functionality, enabling these trial specific requirements to be met.

Aims and objectives

This study aims to qualitatively assess the feasibility and acceptability of the PROmics system, an ePRO platform for use in trials of ATMPs. The planned study will evaluate the use of PROmics when deployed in a trial environment generally and, more specifically, in the POLARISE study, a phase II multi-disease ATMP trial in patients with immune-mediated inflammatory disease.

The specific study objectives are:

1. To explore with PROmics users, their' experiences of being trained with and using the PROmics ePRO system;
2. To explore decision-making with POLARISE trial participants who did not consent to report their PROs via PROmics or withdrew from using the PROmics;
3. To explore the benefits and shortcomings of PROmics and how these may be developed further;
4. To explore how the use of PROmics was managed by research staff and research participants in the context of the POLARISE trial.

METHODS AND ANALYSIS

For peer review only - http://bmjopen.bmj.com/site/about/guidelines.xhtml

This study will be reported in accordance with the Consolidated Criteria for Reporting Qualitative Research (COREQ).[28]

Design

A remote, qualitative, interview-based feasibility study (see figure 1) embedded within the POLARISE trial, a single arm, phase II ATMP basket trial recruiting from six National Health Service (NHS) trusts in the United Kingdom.

Participant selection

In total, it is estimated that 20 to 30 participants will be recruited to this feasibility study. Recruitment will proceed until data saturation, the point in data collection when new data are redundant, is achieved.[29] Two participant groups, POLARISE trial participants (n = 10 – 15 participants) and members of the POLARISE research team (n = 10-15 participants) will be enrolled.

Sampling will be purposive for maximum variation for both POLARISE participants and research staff. Maximum variation where possible will be sought to ensure broad representation of disease groups, age, sex, ethnicity, research site/geographic location (POLARISE participants) and age, sex, clinical/research role, research site/geographic location (research staff).

To take part in the PROmics feasibility study, patients must be enrolled in the POLARISE trial (therefore meeting the trial eligibility criteria) and have either: 1) consented to report their PRO data (symptoms, tolerability, quality of life) using the PROmics ePRO system; 2) consented initially to report their PROs using the PROmics and subsequently withdrew from using the PROmics system to report their PRO data, reverting to paper questionnaires; or 3) declined to report their PROs using the PROmics software. Sampling will aim to recruit between three and five participants from each disease group and any POLARISE participants who decline to use or subsequently withdraw from using the PROmics system.

Participants who are research staff will be members of the research team who have been involved in the planning, delivery, administration and use of the PROmics ePRO system including: 1) research nurses working on the POLARISE trial; 2) POLARISE trial staff; and 3) staff involved in the management of POLARISE trial data. Research staff were enrolled to evaluate the feasibility, acceptability and ease of use of the clinician-facing components of the PROmics system (e.g., clinical dashboard) and to explore perceptions and attitudinal responses to ePRO data capture within an ATMP trial.

All PROmics participants must be able to converse in everyday English and be 18 years old or older. Exclusion criteria include being unable to read, understand, and complete questionnaires presented in English.

Recruitment and consent

All POLARISE patient participants will be eligible to take part in this feasibility study and will be provided with information packs relating to the feasibility study upon enrolment in the POLARISE trial. The first patient will be registered to POLARISE on 19th April 2022 and the trial is estimated to recruit for two years. POLARISE participants will be involved in the trial for approximately 25 months. The estimated duration of the planned feasibility study is dependent on trial recruitment. Interviews will be scheduled with patient participants four to six months after enrolment into the POLARISE trial.

The PROmics feasibility study information packs will be tailored depending on whether a POLARISE participant has: 1) consented to report their PRO data via the PROmics system (accept); 2) consented to provide PRO data via PROmics and changed to paper-based PROs (withdraw); or 3) declined to report their PRO data via the PROmics software (decline). All versions (accept/withdraw/decline) of the information pack will include a cover letter, participant information sheet, and an expression of interest form (+ reply paid envelope) to be returned by the participant to the PROmics research team if wish to

For peer review only - http://bmjopen.bmj.com/site/about/guidelines.xhtml

take part in the feasibility study. POLARISE participants who decline to take part in the feasibility study (i.e., do not return an expression of interest form) will complete the PROs using their preferred method (i.e., using PROmics or completing paper forms) and will have no further contact with the PROmics research team.

POLARISE research team participants will be approached and provided with recruitment packs through liaison with the POLARISE trial sites. The POLARISE research staff recruitment packs will include a cover letter, participant information sheet, and expression of interest form.

A semi-structured interview conducted virtually (e.g., via telephone or videoconference) will be arranged with all participants (patients and members of the POLARISE research team) who express an interest in participating in this feasibility study. Verbal consent will be sought at the time of the telephone/videoconference interview. A pre-specified consent script will enable consent to be taken via the telephone. Participants will be reminded of the study aims and that they are able to withdraw from the study at any time. The consenting process will be completed prior to commencing the interview and will be audio-recorded.

Data collection

Interviews will be arranged at a time point in the POLARISE trial to allow participants sufficient opportunity to use the PROmics system and to ensure accurate recall of their experiences whilst enrolled in the POLARISE trial (i.e., approximately 4-6 months after POLARISE trial enrolment). As the POLARISE trial is a national trial with multiple sites, this method of data collection was selected to enable: 1) ease of communication between participants and the PROmics research team and 2) ensure safe social distancing for COVID-19.

Semi-structured topic guides (see online supplemental file 1) will support patient and research staff interviews and will ensure key topics are consistently covered. The guides will include set/specific questions designed to elicit descriptions of participants' user experiences of the PROmics system, product perception, compliance, acceptability, and feasibility of use. The semi-structured nature of the planned interviews will enable the researcher conducting the interviews to explore topics in depth and accommodate emerging themes through prompts and questions that are informed by the participants' responses. All interviews will be audio-recorded using a digitally encrypted recorder and transcribed verbatim by a qualified, professional transcription service working within a confidentiality agreement. To confirm the accuracy of the interview transcripts, the transcripts will be reviewed against the audio-recording by the PROmics research team. All transcripts will be de-identified and a unique identifier will be assigned to each participant prior to undertaking data analyses. Data collection and analysis will take place concurrently and will continue until the research team agrees data saturation has been reached (per participant group and overall).

Data analysis

The de-identified interview transcripts will be entered into NVivo (Version 20) qualitative data analysis software and analysed using thematic analysis and the framework method described by Gale et al. to identify, describe, and report patterns in the data.[30-32] The framework method is considered appropriate for use with concrete data in studies where research outcomes are clearly defined.[32] Two researchers from the PROmics research team (CM, SEH) will independently code six interview transcripts (three per participant group). Coding will proceed inductively line-by-line to develop a set of initial codes. The researchers will compare codes to produce a consensus-based coding framework which will be checked by the wider research team before being applied across the data set. Codes will be grouped to form categories and categories refined to represent higher-level themes which encompass the full data set and present a clear, coherent and detailed interpretation of the data. Each participant group (i.e., patients and research staff) will be analysed separately and then compared later

For peer review only - http://bmjopen.bmj.com/site/about/guidelines.xhtml

in the analysis. The final analysis will be discussed with the POLARISE trial Patient and Public Involvement and Engagement (PPIE) group to establish the trustworthiness and validity of the findings.

Reflexivity

Reflexivity may be defined as researchers' acknowledgment of their own beliefs, judgements and practices and the role these play during the research process.[33] It is central to conducting a thematic analysis as the researcher plays an active role in the identification patterns within the data.[30, 31] The PROmics research team comprises experts in PROs (MC, SEH, CM), qualitative research (CM, SEH), clinical trials (AR, RM), ePROs (CF, EHD), and a consultant hepatologist (PN). It is nested within the larger POLARISE trial research team. Two experienced qualitative researchers (CM, SEH) will conduct the interviews and undertake data analysis. Reflexive research journals and memo-writing will be used to acknowledge the role of the researcher lens during the analytic process. None of the research team have any relationship with patient participants; however, it is possible that participants who are research staff at POLARISE trial sites may be known contacts to some members of the PROmics team.

Patient and public involvement

PPIE in the PROmics study and wider POLARISE trial was informed by the National Institute for Health Research (NIHR) guidance on the involvement of patient public contributors to research.[34] Patient partners were involved from the outset of the project. To date, PPIE members have informed the design and content of the PROmics ePRO system. A series of stakeholder meetings were convened to establish patient preferences for the format and layout of the PROmics system and to inform PRO selection. Prior to deployment in the POLARISE trial, usability testing of the PROmics system was completed with PPIE members. The outcomes of the stakeholder engagement events and usability testing are reported elsewhere. This protocol was reviewed by patient partner (RW) and the Liver and Gastro-Intestinal PPI Reference Group at the University of Birmingham and the Birmingham Rheumatology Research Patient Partnership (R2P2) who will also support the dissemination of information about the study. Patient and public involvement will be evaluated at the end of the project using the GRIPP-2 checklist.[35, 36]

ETHICS AND DISSEMINATION

Ethical approval for the PROmics feasibility study was granted as part of the POLARISE trial by London - West London & GTAC Research Ethics Committee (21/LO/0475). Local R&D approvals will be obtained prior to recruitment at sites. Informed consent will be obtained verbally and audio-recorded from all participants before data collection. Participants consent to being interviewed and the interviews being audio recorded and transcribed verbatim, and they acknowledge that their words can be used anonymously in the presentation of the research, that they can withdraw their data within seven working days of completing the interview, that participation is voluntary, and data management and storage is subject to the UK General Data Protection Regulation (2018). The results of this study will be disseminated via peer-reviewed journals, conference presentations and shared via social media. The PPIE groups and ATMP stakeholder networks will be consulted to maximise dissemination and impact.

DISCUSSION

Clinical trialists, regulators and policy-makers recognise increasingly the importance of the patient experience in the evaluation of the effectiveness, safety, and tolerability of medicinal products, including in trials of advanced cellular and gene therapies.[2, 37] Accordingly, PRO assessments are included in clinical trials to measure constructs such as symptom severity and functioning and health-related quality of life and, increasingly, these data are collected using ePROs. However, the impact on patients and

For peer review only - http://bmjopen.bmj.com/site/about/guidelines.xhtml

the feasibility and acceptability of ePRO data capture has yet to be established fully in ATMP trials. This qualitative study aims to provide a deeper understanding into the use ePROs in clinical trials of advanced therapies. Barriers and enablers to the use of PROmics, a new ePRO data capture system, will be explored within an ATMP trial for patients with immune-mediated inflammatory disease. These qualitative data will be supplemented with further quantitative findings at the end of the POLARISE trial including compliance and rates of missing data. The findings will inform recommendations to improve the PROmics system, to enhance PRO strategy in future studies; promote efficient use of measures, data completeness, and acceptability to trial participants; inform future analyses and sample size estimation; and support future implementation of ePROs in ATMP trials and routine care.

The study will incorporate the views of a range of stakeholders. The involvement of trial participants from different disease cohorts and from different geographical locations in the UK will enhance variation in the study sample. These patient participants will provide insights into user confidence with digital technology and the completion of PROs electronically, time points of administration, and the overall user experience. Inclusion of trial participants who declined or withdrew from using the PROmics will allow us to explore potential barriers and solutions to participation including inclusion of underserved populations. The views of trial staff and site research nurses will help understand PROmics integration with local IT services and the administrative burden on staff as well as barriers to and enablers of the use of PROmics for data collection, trial participant surveillance, and AE reporting.

The use of qualitative methods is a strength of this study, allowing in-depth exploration of user experience. Nesting the feasibility study within the POLARISE trial will enable timely data collection thereby limiting recall bias. The study sample will include research participants from NHS hospitals from different regions of the UK which may enhance the transferability and generalisability of the study findings. However, as not all trial participants will take part in the feasibility study, sampling is potentially biased. Digital exclusion, particularly for underserved populations that may have limited access to digital devices, contributes a further source of bias to the proposed study.[38] Sampling bias will be addressed by recruiting purposively for maximum variation and through the inclusion of participants who declined to use the PROmics system or who reverted to the use of paper-based PROs at some point in the trial. Questionnaires programmed onto the PROmics system are currently in UK English, however, if the solution proves feasible, inclusion of translated, culturally validated measures will be undertaken in future work.

The number of ATMP trials continues to grow year-on-year, offering potentially powerful new treatments for patients with rare or life-threatening diseases.[39] As with all investigational medicinal products, demonstrating efficacy, safety and tolerability of new ATMPs is crucial. Electronic PROs have the potential to contribute important evidence of efficacy, safety and tolerability (both in the short and longer term) for these novel therapies and to support patient monitoring and risk management. Data collected via the PROmics system has the potential to provide new insights into the reporting of symptomatic AEs specific to the advanced therapies context whilst evaluation of the feasibility and acceptability of the PROmics system will contribute new knowledge to support the development of ePRO reporting formats that meet the unique needs of advanced therapies, for the benefit of all who receive these novel treatments.

Acknowledgments

The authors thank POLARISE Trial Steering Committee, POLARISE Scientific Advisory Group, PROmics Project Management Group, and Patient and Public Involvement (PPI) group members for their input and support of the project.

Contributors

For peer review only - http://bmjopen.bmj.com/site/about/guidelines.xhtml

The feasibility study concept and design were conceived by AR, AmR, PN, MC, RW, RH, GP. CB, AR will support recruitment. EHD, CF, GM were responsible for the development and programming of PROmics in Atom5™. EHD, CF will support POLARISE data collection using the PROmics system. SEH and CM will consent participants and undertake the interviews and data analysis. The protocol was written by AmR, RM, MC, AR, RW, PN. SEH prepared the first draft of the manuscript. AW will provide administrative support to the project. All authors (SEH, CM, AR, AmR, RM, CB, EHD, CF, GM, RH, GP, RW, PN, MC, AW) reviewed the manuscript, provided edits and approved the final version.

Funding

The author(s) disclosed receipt of the following financial support for the research, authorship, and/or publication of this article: This work was supported by the National Institute for Health Research (NIHR) Birmingham Biomedical Research Centre, Midlands-Wales Advanced Therapies Treatment Centre (MW-ATTC) programme grant from Innovate UK to a consortium of partners including Health Technology Wales, the Welsh Blood Service and the University of Birmingham (Grant number: IUK: 104232) and Innovate UK (part of UK Research and Innovation) grant Patient-reported outcomes assessment to support accelerated access to advanced cell and gene therapies: PROmics (Grant No: IUK: 104777). The funder was not involved in any aspect of the research work.

Competing interests

SEH receives funding from the receives funding from the NIHR Oxford-Birmingham Blood and Transplant Research Unit (BTRU) in Precision Therapeutics, UK Research and Innovation (UKRI) and declares personal fees from Cochlear Ltd, Astra Zeneca, and Aparito Ltd. *MC* is a National Institute for Health Research (NIHR) Senior Investigator and receives funding from the National Institute for Health Research (NIHR) Birmingham Biomedical Research Centre, the NIHR Oxford-Birmingham Blood and Transplant Research Unit (BTRU) in Precision Therapeutics, the NIHR Surgical Reconstruction and Microbiology Research Centre and NIHR ARC West Midlands at the University of Birmingham and University Hospitals Birmingham NHS Foundation Trust, Health Data Research UK, UKRI, Macmillan Cancer Support, UCB Pharma Gilead, Janssen and GSK. MC has received personal fees from Astellas, Aparito Ltd, CIS Oncology, Takeda, Merck, Daiichi Sankyo, Glaukos, GSK and the Patient-Centered Outcomes Research Institute (PCORI) outside the submitted work. *CM* receives funding from NIHR Surgical Reconstruction and Microbiology Research Centre (SRMRC), the NIHR Oxford-Birmingham Blood and Transplant Research Unit (BTRU) in Precision Therapeutics UKRI, and declares personal fees from Aparito Ltd outside the submitted work. All other authors have no interests to declare.

Disclaimer

The views expressed are those of the authors and not necessarily those of Innovate UK.

Patient consent for publication

Not required.

Provenance and peer review

Not commissioned, externally peer reviewed.

Open access

This is an open access article distributed in accordance with the Creative Commons Attribution 4.0 International license (CC BY 4.0), which permits others to distribute, remix, adapt, build upon this work, and license their derivative works on different terms, provided the original work is properly cited, appropriate credit is given, and any changes made indicated. See: http:// https://creativecommons.org/licenses/by/4.0/.

REFERENCES

1. Elverum K, Whitman M. Delivering cellular and gene therapies to patients: solutions for realizing the potential of the next generation of medicine. *Gene Ther* 2020;27:537-44. doi: 10.1038/s41434-019-0074-7
2. Lasiter L, Campbell A, Basch E, et al. Use of patient-reported outcomes to understand & measure the patient experience of novel cell and gene therapies. *Ther Innov Regul Sci* 2020;54:1566-75. doi: 10.1007/s43441-020-00184-6

For peer review only - http://bmjopen.bmj.com/site/about/guidelines.xhtml

3. Hollingsworth I. Increasing patient access to advanced therapies. *International Biopharmaceutical Industry* 2020; 3(1). https://ww https://www.theattcnetwork.co.uk/news/increasing-patient-access-to-advanced-therapies-the-uk-perspective.
4. Kyte D, Ives J, Draper H, et al. Current practices in patient-reported outcome (PRO) data collection in clinical trials: a cross-sectional survey of UK trial staff and management. *BMJ Open* 2016;6:e012281. doi: 10.1136/bmjopen-2016-012281
5. FDA. Guidance for industry: Patient-reported outcome measures: use in medicinal product development to support labelling claims, 2009. https://www.fda.gov/regulatory-information/search-fda-guidance-documents/patient-reported-outcome-measures-use-medical-product-development-support-labeling-claims 6. Calvert M, Kyte D, Mercieca-Bebber R, et al. Guidelines for inclusion of patient-reported outcomes in clinical trial protocols: the SPIRIT-PRO Extension. *JAMA* 2018;319:483-94. doi: 10.1001/jama.2017.21903
7. Coons SJ, Eremenco S, Lundy JJ, et al. Capturing patient-reported outcome (pro) data electronically: the past, present, and promise of epro measurement in clinical trials. *Patient* 2015;8:301-09. doi: 10.1007/s40271-014-0090-z
8. Holch P, Pini S, Henry AM, et al. eRAPID electronic patient self-reporting of adverse-events: Patient Information and advice: a pilot study protocol in pelvic radiotherapy. *Pilot Feasibility Stud* 2018;4:110. doi: 10.1186/s40814-018-0304-6
9. Kyte D, Ives J, Draper H, et al. Management of patient-reported outcome (pro) alerts in clinical trials: a cross sectional survey. *PLoS One* 2016;11:e0144658-e58. doi: 10.1371/journal.pone.0144658
10. Basch E, Deal AM, Kris MG, et al. Symptom monitoring with patient-reported outcomes during routine cancer treatment: a randomized controlled trial. *J Clin Oncol* 2016;34:557-65. doi: 10.1200/JCO.2015.63.0830
11. European Medinces Agency. Guideline on Safety and Efficacy Follow-Up - Risk Management of Advanced Therapy Medicinal Products. 2008. https://www.ema.europa.eu/en/documents/scientific-guideline/guideline-safety-efficacy-follow-risk-management-advanced-therapy-medicinal-products_en.pdf.
12. Calvert M, Kyte D, Price G, et al. Maximising the impact of patient reported outcome assessment for patients and society. *BMJ* 2019;364:k5267. doi: 10.1136/bmj.k5267
13. Kluetz PG, Kanapuru B, Lemery S, et al. Informing the Tolerability of Cancer Treatments Using Patient-Reported Outcome Measures: Summary of an FDA and Critical Path Institute Workshop. *Value Health* 2018;21:742-47. doi: https://doi.org/10.1016/j.jval.2017.09.009
14. European Medicines Agency. Appendix 2 to the guideline on the evaluation of anticancer medicinal products in man: The use of patient-reported outcome (PRO) measures in oncology studies. 2016. https://www.ema.europa.eu/en/documents/other/appendix-2-guideline-evaluation-anticancer-medicinal-products-man_en.pdf.
15. Coleman RL, Beck JT, Baranda JC, et al. The use of patient-reported outcome measures in phase i oncology clinical trials. *OCL* 2021;99(7):444-53. doi: 10.1159/000514874
16. Retzer A, Aiyegbusi OL, Rowe A, et al. The value of patient-reported outcomes in early-phase clinical trials. *Nat Med* 2022;28:18-20. doi: 10.1038/s41591-021-01648-4
17. Smith AW, Mitchell SA, K. De Aguiar C, et al. News from the NIH: Person-centered outcomes measurement: NIH-supported measurement systems to evaluate self-assessed health, functional performance, and symptomatic toxicity. *Transl Behav Med* 2016;6:470-74. doi: 10.1007/s13142-015-0345-9
18. Kluetz PG, Chingos DT, Basch EM, et al. Patient-reported outcomes in cancer clinical trials: measuring symptomatic adverse events with the national cancer institute's patient-reported outcomes version of the common terminology criteria for adverse events (PRO-CTCAE). *Am Soc Clin Oncol Educ Book* 2016;36:67-73. doi: 10.1200/EDBK_159514
19. Dueck AC, Mendoza TR, Mitchell SA, et al. Validity and reliability of the us national cancer institute's patient-reported outcomes version of the common terminology criteria for adverse events (PRO-CTCAE). *JAMA Oncol* 2015;1:1051-59. doi: 10.1001/jamaoncol.2015.2639
20. Pearman TP, Beaumont JL, Mroczek D, et al. Validity and usefulness of a single-item measure of patient-reported bother from side effects of cancer therapy. *Cancer* 2018;124:991-97. doi: https://doi.org/10.1002/cncr.31133
21. Herdman M, Gudex C, Lloyd A, et al. Development and preliminary testing of the new five-level version of EQ-5D (EQ-5D-5L). *Qual Life Res* 2011;20:1727-36. doi: 10.1007/s11136-011-9903-x

For peer review only - http://bmjopen.bmj.com/site/about/guidelines.xhtml

22. Cella DF, Tulsky DS, Gray G, et al. The Functional Assessment of Cancer Therapy scale: development and validation of the general measure. *J Clin Oncol* 1993;11:570-79. doi: 10.1200/jco.1993.11.3.570
23. Webster K, Cella D, Yost K. The Functional Assessment of Chronic Illness Therapy (FACIT) Measurement System: properties, applications, and interpretation. *Health Quality Life* 2003;1:79. doi: 10.1186/1477-7525-1-79
24. Krupp LB, LaRocca NG, Muir-Nash J, et al. The Fatigue Severity Scale: application to patients with multiple sclerosis and systemic lupus erythematosus. *Arch Neurol* 1989;46:1121-23. doi: 10.1001/archneur.1989.00520460115022
25. Younossi ZM, Afendy A, Stepanova M, et al. Development and validation of a primary sclerosing cholangitis–specific patient-reported outcomes instrument: the PSC PRO. *Hepatology* 2018;68:155-65. doi: https://doi.org/10.1002/hep.29664
26. CTE Programe. Common Terminology Criteria for Adverse Events (CTCAE) v4.0: NCI; [Available from: https://evs.nci.nih.gov/ftp1/CTCAE/CTCAE_4.03/CTCAE_4.03_2010-06-14_QuickReference_5x7.pdf.
27. David T, Ling SF, Barton A. Genetics of immune-mediated inflammatory diseases. *Clin Exp Immunol* 2018;193:3-12. doi: 10.1111/cei.13101
28. Tong A, Sainsbury P, Craig J. Consolidated criteria for reporting qualitative research (COREQ): a 32-item checklist for interviews and focus groups. *Int J Qual Health Care* 2007;19:349-57. doi: 10.1093/intqhc/mzm042
29. Saunders B, Sim J, Kingstone T, et al. Saturation in qualitative research: exploring its conceptualization and operationalization. *Qual Quant* 2018;52:1893-907. doi: 10.1007/s11135-017-0574-8
30. Braun V, Clarke V. Using thematic analysis in psychology. *Qual Res Psychol* 2006;3:77-101. doi: 10.1191/1478088706qp063oa
31. Braun V, Clarke V. Reflecting on reflexive thematic analysis. *Quali Res Sport, Exerc Health* 2019;11:589-97. doi: 10.1080/2159676X.2019.1628806
32. Gale NK, Heath G, Cameron E, et al. Using the framework method for the analysis of qualitative data in multi-disciplinary health research. *BMC Med Res Methodol* 2013;13:117. doi: 10.1186/1471-2288-13-117
33. Finlay L. Reflexivity: An essential component for all research? *Br J Occup Ther* 1998;61:453-56. doi: 10.1177/030802269806101005
34. National Institute for Health Research. Centre for Dissemination and Engagement recognition payments for public contributors 2020. Available from: https://www.nihr.ac.uk/documents/centre-for-engagement-and-dissemination-recognition-payments-for-public-contributors/24979.
35. Staniszewska S, Brett J, Simera I, et al. GRIPP2 reporting checklists: tools to improve reporting of patient and public involvement in research. *Res Involv Engagem* 2017;3:13. doi: 10.1186/s40900-017-0062-2
36. Kok M, Gibson A, Evans D, et al. Guidance document: evaluating public involvement in research. 2018. http://www.phwe.org.uk/wp-content/uploads/Guidance-on-evaluating-Public-Involvement-in-research.pdf.
37. Chakraborty R, Sidana S, Shah GL, et al. Patient-reported outcomes with chimeric antigen receptor t cell therapy: challenges and opportunities. *Biol Blood Marrow Transplant* 2019;25:e155-e62. doi: https://doi.org/10.1016/j.bbmt.2018.11.025
38. Calvert MJ, Cruz Rivera S, Retzer A, et al. Patient reported outcome assessment must be inclusive and equitable. *Nat Med* 2022;28:1120-24. doi: 10.1038/s41591-022-01781-8
39. Hanna E, Rémuzat C, Auquier P, et al. Advanced therapy medicinal products: current and future perspectives. *J Mark Access Health Policy* 2016;4:10.3402/jmahp.v4.31036. doi: 10.3402/jmahp.v4.31036

FIGURE TITLE

Figure 1. PROmics qualitative feasibility study embedded within the POLARISE trial—participant flowchart

For peer review only - http://bmjopen.bmj.com/site/about/guidelines.xhtml

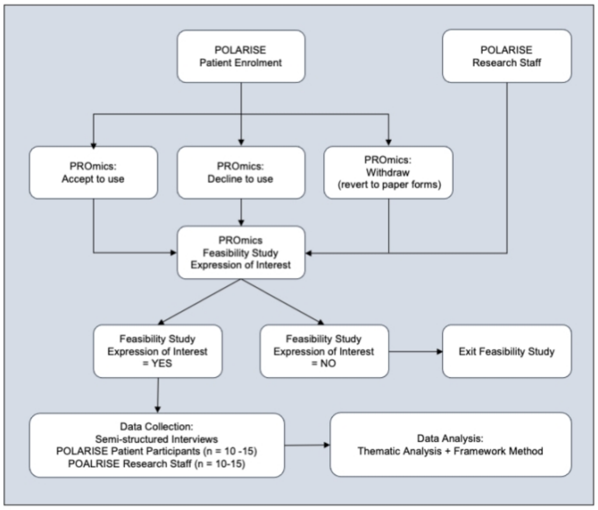

Figure 1. PROmics qualitative feasibility study embedded within the POLARISE trial. Flow chart of participants.

107x60mm (300 x 300 DPI)

SUPPLEMENTAL FILE 1 – INTERVIEW TOPIC GUIDE

PATIENTS

1. Restate the purpose of the interview: *Discuss PROmics system*
2. Select the interview structure based on patient's ACCEPT, WITHDRAW, DECLINE status: If ACCEPT, ask ACCEPT questions only. If WITHDRAW, ask ACCEPT and WITHDRAW questions. If DECLINE, ask DECLINE questions only.
3. Any questions or clarification before beginning? Feel free to take a break at any point during the interview.
4. Materials: paper questionnaires, app with PROmics software for reference

ACCEPT

1. Why did you choose to use the PROmics software?
- Was there anything that made you question if you should use it? (FURTHER PROBES: Burden, confidence with technology)
- Were you given enough information?
2. Did you find the initial training with the PROmics software helpful?
- Was there anything particularly good or bad about the training?
- How could it be improved?
3. How did you find using the PROmics software?
- Did you feel like you had enough support?
- How could the support for patients using the PROmics software be improved?
4. How did use of the PROmics software fit in with your other trial-related activities? (FURTHER PROBE: Burden)
- Did it make you feel more or less engaged with the trial overall? How?
- Did it make you feel like you were getting better or worse care during the trial? How?
5. How did you find the PROmics software to use?
- What did you like? Why?
- What didn't you like? Why?
- How could it be improved?
- How does it compare to paper questionnaires? (SHOW: Paper questionnaires)
6. Would you recommend for others to use the PROmics software if they participating in a trial? Why?

WITHDRAW

1. Why did you decide to stop using the PROmics software?
- What could have been done to help you continue to use the software?

DECLINE

1. Why did you choose not to use the PROmics software?

For peer review only - http://bmjopen.bmj.com/site/about/guidelines.xhtml

UNIVERSITY OF BIRMINGHAM | CPROR MAKING PATIENT CENTRED CARE A REALITY

- Was there anything that made you question if you should use it? (FURTHER PROBES: Burden, confidence with technology)
- Were you given enough information?

2. How does the PROmics software compare to paper questionnaires? (SHOW: app)

- Do you think you would use the PROmics software if you were in a trial again?

RESEARCH TEAM

1. Restate the purpose of the interview: *Discuss PROmics system*
2. Select the interview structure based on participant's experience: Site Research Nurse/ other. If OTHER, ask OTHER questions only. If SITE RESEARCH NURSE, ask SITE RESEARCH NURSE and OTHER questions.
3. Any questions or clarification before beginning? Feel free to take a break at any point during the interview.
4. Materials: paper questionnaires, demo iPad with PROmics software for reference

SITE RESEARCH NURSE

1. Did you find the initial training with the PROmics software helpful?

- Was there anything particularly good or bad about the training?
- How could it be improved?
- Were you given enough information?

2. How did you find using the PROmics software?

- Did you feel like you had enough support?
- How could the support for staff using the PROmics software be improved?

3. How did you find the PROmics software to use?

- What did you like? Why?
- What didn't you like? Why?
- How could it be improved?
- How does it compare to paper questionnaires? (SHOW: Paper questionnaires)

4. How did using the PROmics software impact on your care of patients?

- Was it better/worse?
- How could this be improved?

5. Did you feel equipped to train patients to use the PROmics software?

- How could this be improved?

6. Did you feel equipped to support patients to use the PROmics software during the trial?

- How could this be improved?

OTHER

1. How did use of the PROmics software fit in with your other trial-related activities? (FURTHER PROBES: Burden, workload)

- Was there anything that made you question if it should be used in the trial? (FURTHER PROBES: Burden, confidence with technology)
- Would you recommend that the PROmics software is used in future trials?

For peer review only - http://bmjopen.bmj.com/site/about/guidelines.xhtml
